# An immune dysfunction score for stratification of patients with acute infection based on whole blood gene expression

**DOI:** 10.1126/scitranslmed.abq4433

**Published:** 2022-11-02

**Authors:** Eddie Cano-Gamez, Katie L Burnham, Cyndi Goh, Alice Allcock, Zunaira H. Malick, Lauren Overend, Andrew Kwok, David A. Smith, Hessel Peters-Sengers, David Antcliffe, GAinS Investigators, Nigel Webster, Helen Galley, Jane Taylor, Sally Hall, Jenni Addison, Sian Roughton, Heather Tennant, Achyut Guleri, Natalia Waddington, Dilshan Arawwawala, John Durcan, Alasdair Short, Karen Swan, Sarah Williams, Susan Smolen, Christine Mitchell-Inwang, Tony Gordon, Emily Errington, Maie Templeton, Pyda Venatesh, Geraldine Ward, Marie McCauley, Simon Baudouin, Charley Higham, Jasmeet Soar, Sally Grier, Elaine Hall, Stephen Brett, David Kitson, Robert Wilson, Laura Mountford, Juan Moreno, Peter Hall, Jackie Hewlett, Stuart McKechnie, Christopher Garrard, Julian Millo, Duncan Young, Paula Hutton, Penny Parsons, Alex Smiths, Roser Faras-Arraya, Jasmeet Soar, Parizade Raymode, Jonathan Thompson, Sarah Bowrey, Sandra Kazembe, Natalie Rich, Prem Andreou, Dawn Hales, Emma Roberts, Simon Fletcher, Melissa Rosbergen, Georgina Glister, Jeronimo Moreno Cuesta, Julian Bion, Joanne Millar, Elsa Jane Perry, Heather Willis, Natalie Mitchell, Sebastian Ruel, Ronald Carrera, Jude Wilde, Annette Nilson, Sarah Lees, Atul Kapila, Nicola Jacques, Jane Atkinson, Abby Brown, Heather Prowse, Anton Krige, Martin Bland, Lynne Bullock, Donna Harrison, Gary Mills, John Humphreys, Kelsey Armitage, Shond Laha, Jacqueline Baldwin, Angela Walsh, Nicola Doherty, Stephen Drage, Laura Ortiz-Ruiz de Gordoa, Sarah Lowes, Charley Higham, Helen Walsh, Verity Calder, Catherine Swan, Heather Payne, David Higgins, Sarah Andrews, Sarah Mappleback, Charles Hinds, Chris Garrard, D Watson, Eleanor McLees, Alice Purdy, Martin Stotz, Adaeze Ochelli-Okpue, Stephen Bonner, Iain Whitehead, Keith Hugil, Victoria Goodridge, Louisa Cawthor, Martin Kuper, Sheik Pahary, Geoffrey Bellingan, Richard Marshall, Hugh Montgomery, Jung Hyun Ryu, Georgia Bercades, Susan Boluda, Andrew Bentley, Katie Mccalman, Fiona Jefferies, Julian Knight, Emma Davenport, Katie Burnham, Narelle Maugeri, Jayachandran Radhakrishnan, Yuxin Mi, Alice Allcock, Cyndi Goh, Stuart McKechnie, Brendon P. Scicluna, Tom van der Poll, Anthony C. Gordon, Charles J. Hinds, Emma E. Davenport, Julian C. Knight

**Affiliations:** 1 Wellcome Centre for Human Genetics, University of Oxford; Oxford, OX3 7BN, UK; 2 Wellcome Sanger Institute, Wellcome Genome Campus; Cambridge, CB10 1SA, UK; 3 The Jenner Institute, University of Oxford; Oxford, OX3 7DQ, UK; 4 Chinese Academy of Medical Science Oxford Institute, University of Oxford; Oxford, OX3 7BN, UK; 5 Centre for Experimental and Molecular Medicine, Amsterdam University Medical Centers, University of Amsterdam; 1100 DD Amsterdam Southeast, Netherlands; 6 Department of Epidemiology and Data Science, Amsterdam Public Health, Amsterdam University Medical Centers, University of Amsterdam, 1100 DD Amsterdam Southeast, Netherlands; 7 The Amsterdam Institute for Infection and Immunity, Amsterdam University Medical Centers, 1100 DD Amsterdam Southeast, Netherlands; 8 Division of Anaesthesia, Pain Medicine and Intensive Care, Department of Surgery and Cancer, Faculty of Medicine, Imperial College London; London, SW7 2AZ, UK; 9 Oxford University Hospitals NHS Foundation Trust; Oxford, OX3 9DU, UK; 10 Department of Applied Biomedical Science, Faculty of Health Sciences, Materi Dei Hospital, University of Malta; Msida, MSD 2080, Malta; 11 Centre for Molecular Medicine and Biobanking, University of Malta; Msida, MSD 2080, Malta; 12 William Harvey Research Institute, Barts and The London School of Medicine and Dentistry, Queen Mary University; London, EC1M 6BQ, UK; 13 Aberdeen Royal Infirmary, Aberdeen, AB25 2ZN, UK; 14 Blackpool Victoria Hospital, Blackpool, FY3 8NR, UK; 15 Broomfield Hospital, Chelmsford, CM1 7ET, UK; 16 Charing Cross Hospital, London, W6 8RF, UK; 17 Coventry and Warwickshire University Hospital, Coventry, CV2 2DX, UK; 18 Freeman Hospital, Newcastle upon Tyne, NE7 7DN, UK; 19 Frenchay Hospital, Bristol, UK and Southmead Hospital, Bristol, BS16 1JE, UK; s Hammersmith Hospital, London, W12 0HS, UK; 21 Huddersfield Royal Infirmary, Huddersfield, HD3 3EA, UK; 22 John Radcliffe Hospital, Headington, Oxford, OX3 9DU, UK; 23 Kettering General Hospital, Kettering, NN16 8UZ, UK; 24 Leicester Royal Infirmary, Leicester, LE1 5WW, UK; 25 Norfolk and Norwich University Hospital, Norwich, NR4 7UY, UK; 26 North Middlesex Hospital, London, N18 1QX, UK; 27 Queen Elizabeth Hospital, Birmingham, B15 2GW, UK; 28 Royal Berkshire Hospital, Reading, RG1 5AN, UK; 29 Royal Blackburn Hospital, Blackburn, BB2 3HH, UK; 30 Royal Hallamshire Hospital, Sheffield, S10 2JF, UK; 31 Northern General Hospital, Sheffield, S5 7AU, UK; 32 Royal Preston Hospital, Preston, PR2 9HT, UK; 33 Royal Sussex County Hospital, Brighton, BN2 5BE, UK; 34 Royal Victoria Infirmary, Newcastle upon Tyne, NE1 4LP, UK; 35 Southend Hospital, Westcliff-on-Sea, SS0 0RY, UK; 36 St Bartholomew’s Hospital, London, EC1A 7BE, UK; 37 Royal London Hospital, London, E1 1FR, UK; 38 St Mary’s Hospital, London, W2 1NY, UK; 39 The James Cook University Hospital, Middlesbrough, TS4 3BW, UK; 40 The Whittington Hospital, London, N19 5NF, UK; 41 University College London Hospital, UCLH, London, NW1 2BU, UK; 42 Wythenshawe Hospital, Manchester, M23 9LT, UK

## Abstract

Dysregulated host responses to infection can lead to organ dysfunction and sepsis, causing millions of global deaths each year. To alleviate this burden, improved prognostication and biomarkers of response are urgently needed. We investigated the use of whole blood transcriptomics for stratification of patients with severe infection by integrating data from 3,149 samples from patients with sepsis due to community-acquired pneumonia or fecal peritonitis admitted to intensive care and healthy individuals into a gene expression reference map. We used this map to derive a quantitative sepsis response signature (SRSq) score reflective of immune dysfunction and predictive of clinical outcomes, which can be estimated using a 7- or a 12-gene signature. Last, we built a machine learning framework, SepstratifieR, to deploy SRSq in adult and pediatric bacterial and viral sepsis, H1N1 influenza, and COVID-19, demonstrating clinically relevant stratification across diseases and revealing some of the physiological alterations linking immune dysregulation to mortality. Our method enables early identification of individuals with dysfunctional immune profiles, bringing us closer to precision medicine in infection.

## Introduction

Infectious diseases result in considerable global morbidity and mortality (1), and can put individuals at risk of critical illness. In extreme cases, this leads to sepsis, a dysregulated host response characterized by major organ dysfunction which accounts for 11 million yearly deaths (2, 3). Timely identification of dysfunctional patient profiles which are amenable to interventions is therefore fundamental.

High-throughput technologies can be used to stratify individuals by molecular characteristics (4). In sepsis, patient subphenotypes (subgroups) have been described using whole blood gene expression (5–9) or clinical variables (10) in both adult and pediatric populations (11–13). However, the pathophysiological mechanisms underlying these subgroups remain unresolved. We previously described two sepsis response signature (SRS) groups (5): SRS1, an immunocompromised profile showing increased risk of death, and SRS2, an immunocompetent profile with reduced mortality, which may be harmed by corticosteroid treatment (14). However, similar developments are lacking for the wider population of patients with infection who do not fulfill sepsis criteria. Moreover, it is unclear how such information can be used to stratify patients at point-of-care.

We developed SepstratifieR, a machine learning framework which addresses these limitations. SepstratifieR was trained on data from patients with sepsis and healthy individuals encompassing three technological platforms, making it a flexible framework which is robust to technological differences and amenable to point-of-care testing.

SepstratifieR achieves personalized risk prediction by deriving a score reflective of each patient’s extent of immune dysfunction. We show that this score accurately models disease heterogeneity and advances outcome prediction, demonstrating applicability in bacterial and viral sepsis, influenza, and COVID-19.

## Results

### A cross-platform transcriptional map of the host response in sepsis

**Fig. 1:**
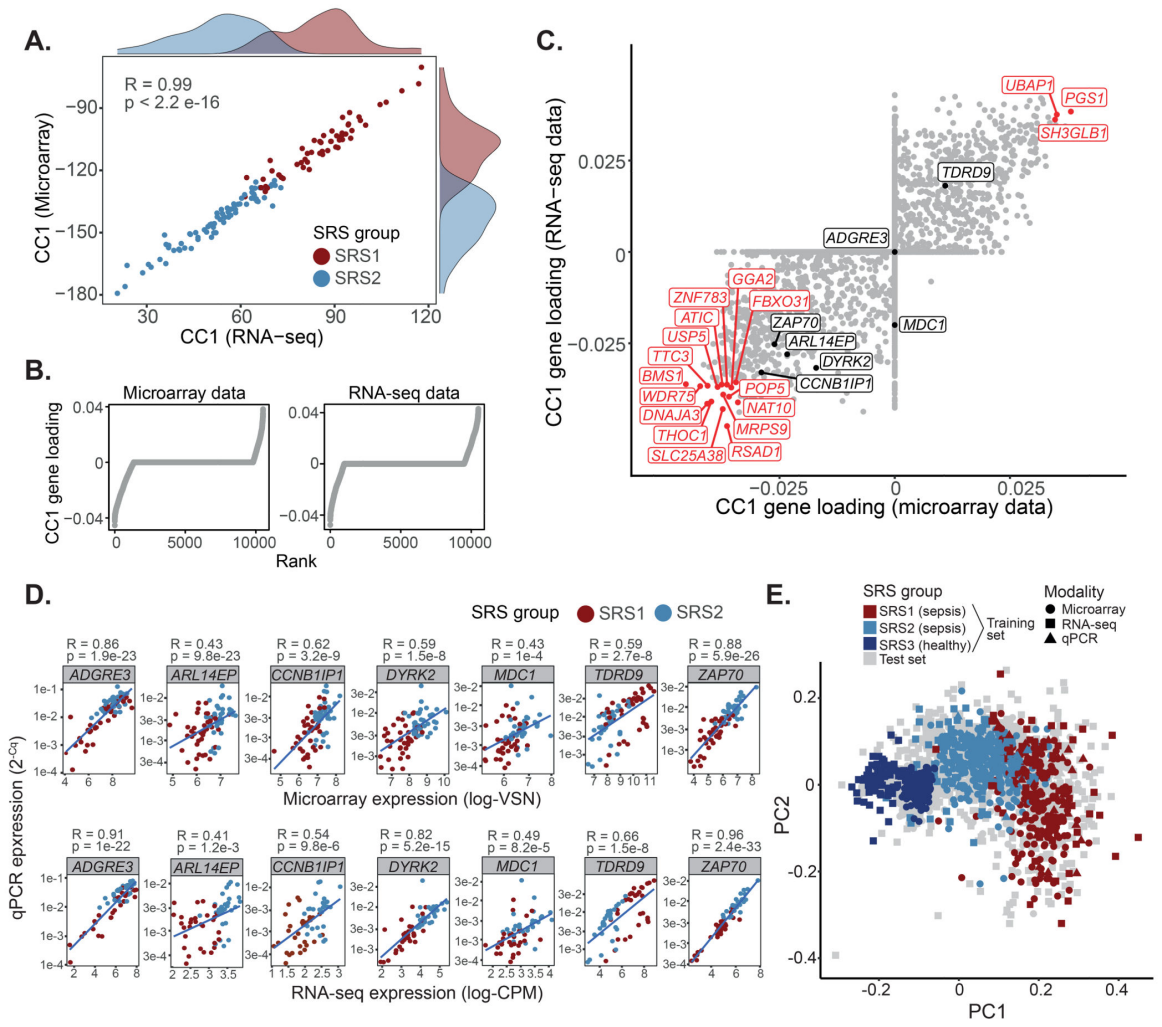
Construction of a reference map of gene expression in sepsis using data from three different platforms. (**A**) CCA analysis of GAinS samples with RNA-seq and microarray data available. Histograms represent marginal SRS1 (red) and SRS2 (blue) distributions. R = Pearson correlation; p = correlation p value. (**B**) Contribution of each gene to CC1, ranked increasingly. (**C**) CC1 contribution of each microarray (X axis) and RNA-seq (Y axis) feature. Black and red dots indicate genes in the Davenport signature and amongst the top 1% highest CC1 contributors, respectively. (**D**) Correlation of microarray/RNA-seq (X axis) and qRT-PCR (Y axis) measurements. Best linear fits are shown. R = Pearson correlation; p = correlation p value. (**E**) A reference map of sepsis based on the Davenport signature (PCA visualization). Dots represent samples, with shapes indicating profiling platform and colors SRS group.

We previously described SRS patient subgroups identified from unsupervised hierarchical clustering of global gene expression of peripheral blood leucocytes using microarrays (5–8). However, it is unclear whether they generalize to sequencing-based assays. Thus, we asked if SRS groups were detectable using RNA-seq by leveraging data from 134 patients from the UK Genomic Advances in Sepsis (GAinS) study with both microarray and RNA-seq measurements available for the total leukocyte population from whole blood samples. We used canonical correlation analysis (CCA) to create a joint representation of both assays. CCA identifies linear combinations of variables (canonical dimensions) that maximize the correlation between two data sets, represented as shared axes of variation. Using SRS assignment known from our previous studies (5, 6), we demonstrated that the first canonical dimension (CC1) separated SRS1 from SRS2 patients (Fig. 1A), indicating that SRS groups are identifiable using RNA-seq.

We previously proposed a 7-gene signature predictive of SRS (5, 6). We now asked whether this signature performed well using RNA-seq data by assessing the contribution of these genes to CC1 (Fig. 1B). We observed non-zero contributions for 6 out of 7 genes (Fig. 1C), demonstrating this signature was applicable to both microarray and RNA-seq data. To assess if the signature was also compatible with rapid turn-around methods, we used quantitative reverse transcription polymerase chain reaction (qRT-PCR) to profile these genes in 115 patients with either microarray or RNA-seq measurements available (Table S1). We observed a significant agreement between methods (P ≤ 0.001 for all tested genes, Fig. 1D), suggesting that our signature might be used for qRT-PCR-based point-of-care testing.

A limitation of this signature is its bias towards SRS2-associated genes. Including more genes could make predictions more resilient. Therefore, we combined this signature with 12 additional genes we identified as ranked amongst the top 1% with highest CC1 contribution (Fig. 1C). This resulted in a 19-gene signature, with all additional genes showing comparable expression to the original gene set (Fig. S1A). We refer to the 7-gene set as the Davenport signature and to the 19-gene set as the Extended signature.

We next compiled data from 1,044 GAinS patients (corresponding to 1,655 whole blood samples) in which total leukocyte gene expression was profiled with up to three platforms, and integrated them based on these signatures, generating cross-technology maps of gene expression in sepsis. To make maps representative of a wider patient population, we also included healthy individuals from three cohorts (Table S1). Integration was performed using a method borrowed from single-cell omics that matched samples in one batch to their nearest neighbors in other batches (15). This resulted in two reference maps: the Davenport map, containing 3,264 samples (1,655 sepsis and 1,609 healthy) and seven genes; and the Extended map, containing 3,149 samples (1,540 sepsis and 1,609 healthy) and 19 genes. Samples in both reference maps clustered by SRS rather than technology, with the main axis of variation showing separation between the healthy volunteer, SRS1, and SRS2 groups (Fig. 1E). Thus, our reference maps capture a wide spectrum of transcriptional variation spanning health and critical illness.

### A classifier model for stratification of patients with sepsis

**Fig. 2:**
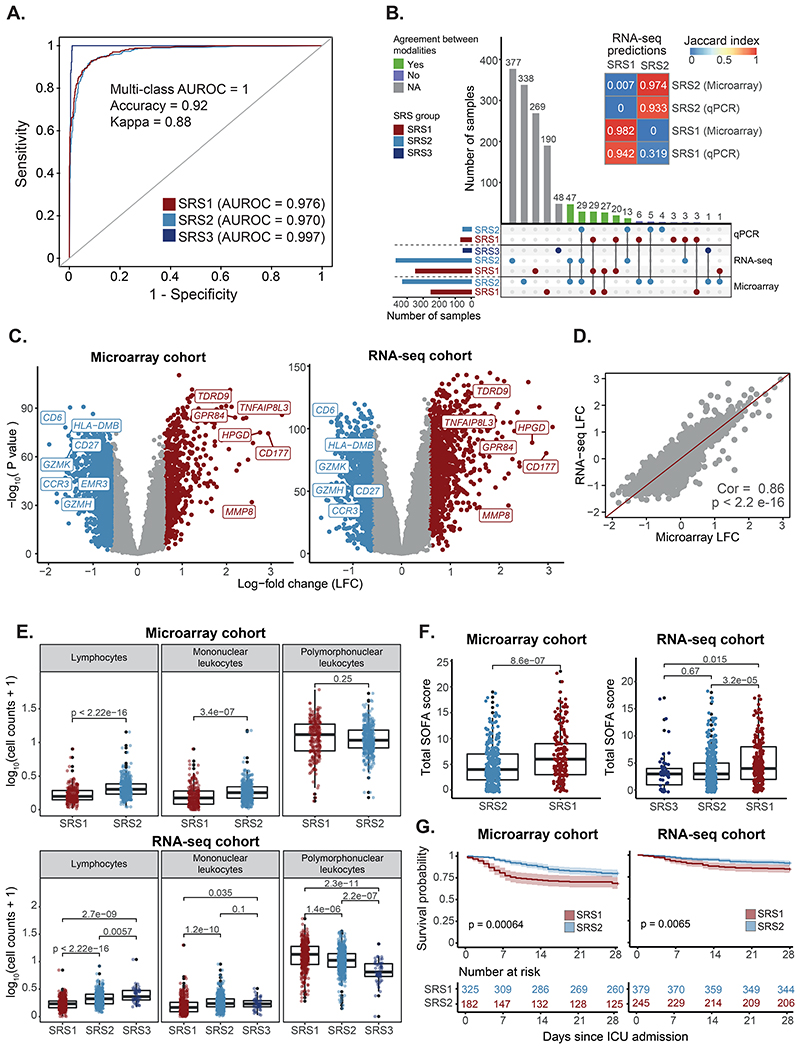
Stratification of patients with sepsis based on whole blood gene expression. (**A**) Receiver operating characteristic (ROC) curves showing cross-validation performance. AUROCs = area under the ROC curve. (**B**) UpSet plot showing prediction agreement between modalities. Colors indicate SRS classes (horizontal) and cross-modality agreement (vertical). Gray bars indicate samples with only one modality available. The heatmap (top) shows the level of cross-modality agreement (Jaccard index). (**C**) Volcano plot showing SRS1/SRS2 differential gene expression. Red indicates upregulation in SRS1 and blue upregulation in SRS2. (**D**) Correlation between SRS-associated log-fold changes from microarray and RNA-seq. The identity line is shown as a reference. Cor = Pearson correlation; p = correlation p value. (**E**) Cell count distribution per SRS group. p = T-test (top) or Kruskal-Wallis (bottom) p value. (**F**) SOFA score distribution per SRS group at the latest available time point. p = T-test (left) or Kruskal-Wallis (right) p value. (**G**) Kaplan-Meier curves of 28-day survival per SRS group, defined at the latest available time point. Shades indicate 95% confidence intervals. p = log-rank test p value.

We next built models for SRS prediction by splitting our reference maps into training (n=909) and test (n=2,355) sets, and training random forest classifiers. Training sets were designed to contain all patient samples taken at ICU admission for which SRS membership was known (n=639), as well as 270 randomly selected healthy volunteer samples. All remaining samples were allocated to the test set. Healthy volunteers were used to define an additional SRS3 group, designed to capture individuals in the low severity/recovery spectrum (that is, transcriptionally closer to health). Cross-validation revealed high accuracy across all SRS groups (AUROCs > 0.97; Fig. 2A), which was confirmed in the test set based on comparisons with previously proposed SRS labels for these samples (5, 6) (accuracy = 0.92 and 0.95 for the Davenport and extended signatures, respectively). Both signatures reached a consensus for the majority of samples (97% and 84% agreement in microarray and RNA-seq, respectively; Fig. S1B), with predictions being consistent across technologies and most samples being assigned to the same SRS group regardless of the profiling method used (Fig. 2B). Thus, our models enable high classification accuracy and cross-technology applicability.

Nonetheless, there are no gold standard SRS labels for these patients, which makes accuracy estimation challenging. Consequently, we further validated these results by testing for differences in biological pathway activity and clinical outcomes. We observed upregulation of neutrophil genes (*MMP8*, *GPR84*, and *CD177*) and downregulation of T cell genes (*CD27*, *CD6*, *CCR3*; Fig. 2C-D) in SRS1, with the top SRS1-associated pathways being Toll-like receptor (TLR) signaling, cytokine production, and glycolysis. In contrast, SRS2 was associated with T cell receptor (TCR) engagement, CD28-costimulation, and IFNγ signaling (Fig. S2A). This was supported by decreased lymphocyte and increased polymorphonuclear cell counts in SRS1 (Fig. 2E). Clinically, SRS1 patients showed higher Sequential Organ Failure Assessment (SOFA) scores, indicative of more severe organ dysfunction in both the microarray and the RNA-seq group (Fig. 2F), as well as increased Acute Physiology and Chronic Health Evaluation (APACHE) II scores in the RNA-seq cohort (Fig. S2B). Survival analysis revealed that, despite the microarray cohort being enriched for non-survivors, SRS1 patients were at an increased risk of death in both cohorts (33% vs 20% mortality, and 16% vs 9% mortality in microarray and RNA-seq, respectively) (Fig. 2G). This demonstrates that our models can successfully predict poor outcome risk.

### A quantitative score reflective of immune dysfunction

**Fig. 3:**
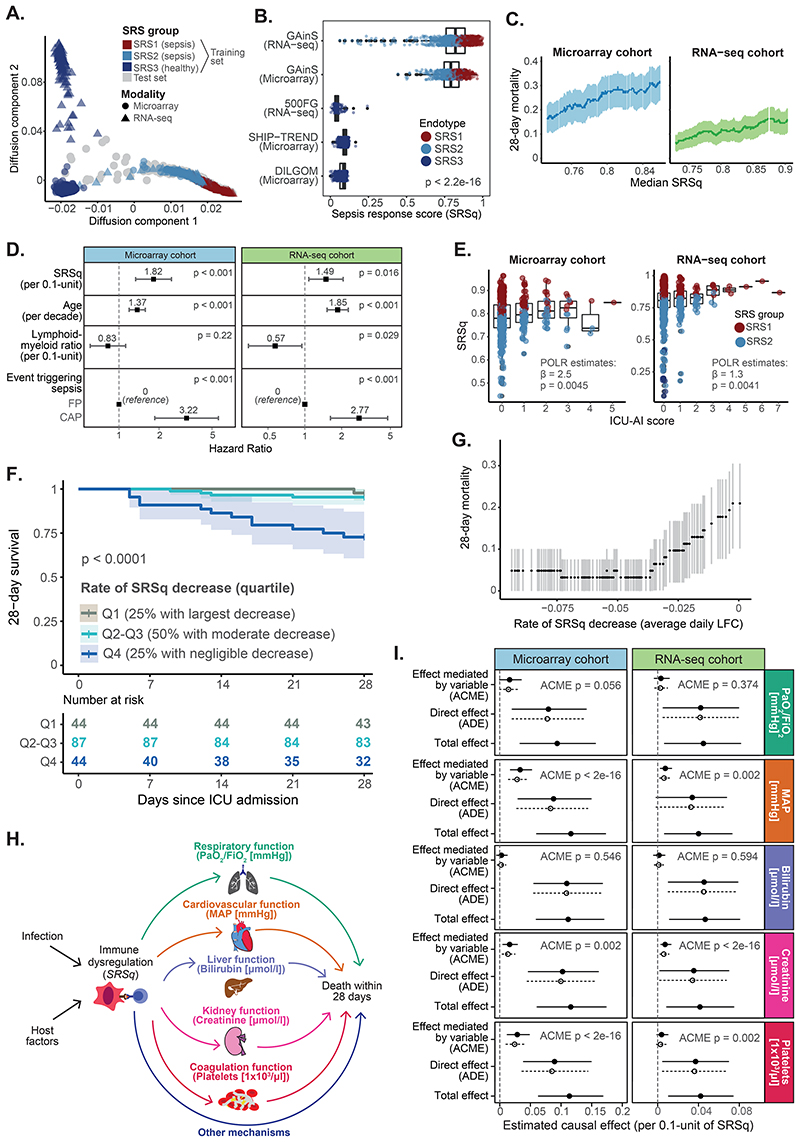
A quantitative score reflective of immune dysfunction severity. (**A**) Diffusion map estimated using the Extended gene signature. Colors indicate SRS group; shapes indicate profiling platforms. (**B**) Distribution of SRSq across cohorts. p = Kruskal-Wallis test p value. (**C**) Association between SRSq and mortality in GAinS, as determined using a sliding window approach. Shades represent 95% confidence intervals. (**D**) Estimated hazard ratios and 95% confidence intervals. (**E**) SRSq values stratified by ICU-acquired infection score (ICU-AI). β = regression coefficient; p = regression p value. (**F**) Kaplan-Meier curves of 28-day survival in patients sampled at multiple time points. Patients were stratified into quartiles based on their rate of SRSq reduction over time. Shades indicate 95% confidence intervals. p = log-rank test p value. (**G**) Association between rate of SRSq reduction and mortality, as determined using a sliding window. Shades represent 95% confidence intervals. (**H**) Causal model assumed for mediation analysis. Arrows represent causal directions. (**I**) Mediation effects. Lines indicate 95% confidence intervals, with solid and dotted lines corresponding to the treatment (high SRSq) and control (low SRSq) conditions. ACME = Average Causal Mediation Effect; ADE = Average Direct Effect; p = mediation p value.

Sepsis encompasses a spectrum of illnesses with varying severities (16, 17), raising the possibility of treating patients as a continuum. Therefore, we used diffusion maps to order the samples in our reference sets into a progression. This separated samples into a continuum which started at SRS3 and gradually transitioned towards SRS2 and SRS1, independently of the technology used for sample profiling (Fig. 3A and Fig. S3A). We used the first diffusion component to derive a quantitative metric reflective of the position of individuals along this continuum, which we refer to as the *quantitative sepsis response signature score* (SRSq). SRSq is bound between 0 and 1, with lower values indicating a patient is transcriptionally closer to health and higher values indicating similarity to the most severe form of sepsis (Fig. 3B). SRSq scores derived using both gene signatures were highly correlated (Pearson correlation = 0.84; P < 2.2e-16). However, the extended signature achieved better separation of patients from controls (Fig. S3B). This suggests that the transcriptomic host response to infection can be modeled as a continuum. Last, to make its calculation more straightforward, we built machine learning models to predict SRSq. We subdivided samples into training and test sets (as defined above) and trained random forest prediction models. Model performance was high in both cross-validation and the test set (RMSE = 0.028; Fig. S3C).

We next investigated the molecular and clinical changes underlying SRSq. Both gene expression (Fig. S3D-E) and cell counts (Fig. S3F) changed along SRSq. Although both SRS and SRSq captured similar gene expression programs (Fig. S3E), our analysis identified 4,121 additional SRSq-associated genes which were not significantly different between SRS groups at the significance threshold of fold-change > 1.5 and FDR < 0.05 (Fig. S3E). This doubled the associated gene set, demonstrating the power of modeling disease as a continuum. From a clinical standpoint, SRSq was significantly associated with 28-day mortality (Fig. 3C; log-rank test P < 0.001 and P = 0.016 for the microarray and the RNA-seq cohort, respectively). A 0.1-unit increase in SRSq was found to decrease patient survival by approximately as much as if the patient were a decade older (HR = 2 and 1.6 in microarray and RNA-seq, respectively). This was true even when accounting for age, source of sepsis, and the lymphoid-to-myeloid cell count ratios (Fig. 3D), suggesting that SRSq goes beyond differences in cellular composition and demographic risk factors. Additionally, SRSq associated with the severity of secondary ICU-acquired infections (Fig. 3E and Table S2). This illustrates the value of SRSq in risk estimation.

We previously reported that patients can change SRS group over time (6). Therefore, we leveraged SRSq to study changes in immune risk over time in 177 patients profiled repeatedly at up to three time points (1st, 3rd, or 5th day in ICU). Of these patients, 80% showed a decrease in SRSq over time, suggesting that this variable captures processes occurring during acute illness. When ranked by the magnitude of SRSq change, patients with the largest decreases in SRSq showed the lowest mortality rates. In contrast, patients with negligible or no SRSq decrease were at a significantly increased risk of death (Fig. 3F). This demonstrates that SRSq is a suitable metric for monitoring illness progression over time.

Last, we investigated the processes linking immune dysfunction and death. Mediation analysis is a statistical tool which tests the compatibility of a hypothesis with existing data by simulating how the response variable would change if other variables were altered one at a time (18, 19). This is roughly equivalent to a computational randomized experiment. We used mediation analysis to test a model where SRSq influences organ dysfunction (SOFA), in turn increasing mortality (Fig. S3G). This enabled us to estimate both direct effects (the expected increase in mortality if SRSq were artificially increased, but SOFA were held constant), and mediation effects (the expected increase in mortality if SOFA were artificially increased, but SRSq were kept constant). This analysis confirmed that the effect of SRSq on mortality was mediated by organ dysfunction (Fig. S3H). We next assessed the role of individual organ dysfunctions by performing mediation analysis on all clinical variables that contribute to SOFA. The effect of SRSq on death was mediated by alterations in arterial pressure, coagulation, and renal function (Fig. 3H-I). In contrast, we found no evidence of liver or lung dysfunction mediating this effect (Fig. 3I and Fig. S3I), despite the large proportion of patients with pneumonia in our cohort. This suggests that, in patients with already severe respiratory infection, systemic consequences of maladaptive inflammation have a larger influence in mortality than lung dysfunction.

### SepstratifieR: a machine learning framework for patient stratification

**Fig. 4:**
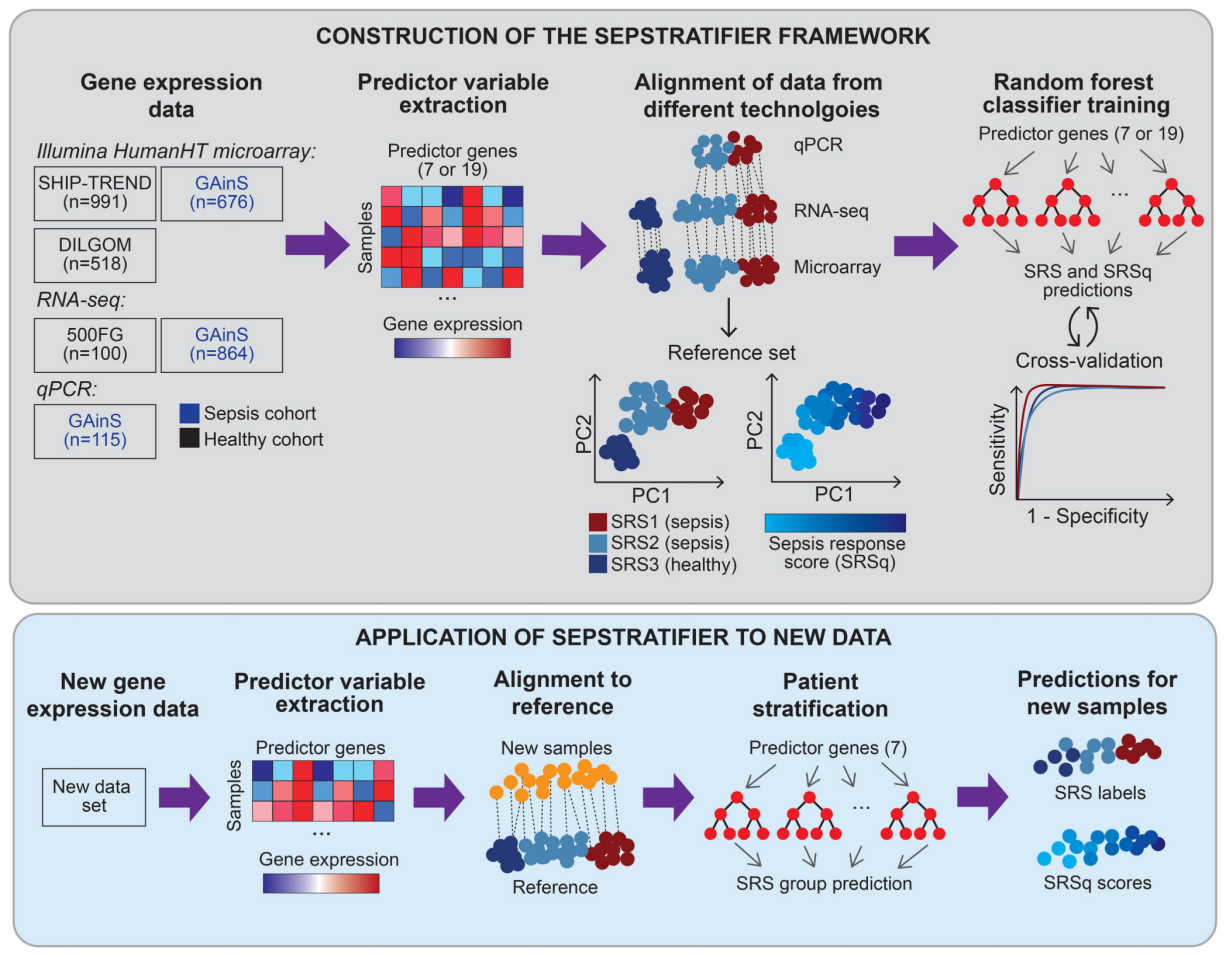
SepstratifieR’s construction and application to new data. Schematic representation of how SepstratifieR was built (top panel) and how it is applied to new data (bottom panel). Publicly available data (5, 6) were used to construct sepsis reference maps based on small gene signatures. Next, random forest models were trained to predict SRS and SRSq. When applying SepstratifieR to new samples, genes in the signature of interest are extracted and used to align new samples to the reference map. After alignment, SRS and SRSq were predicted using pre-trained models.

To make patient stratification accessible, we collected the models described above into an algorithmic framework called SepstratifieR, which extracts the expression values of signature genes, aligns samples to our sepsis reference maps, and predicts SRS and SRSq using random forest models (Fig. 4). This can be achieved using a single line of code (https://github.com/jknightlab/SepstratifieR). We assessed the stability of this algorithm at different sample sizes by testing it in subsets of patients from an independent cohort (20). SepstratifieR remained accurate at sample sizes as low as 20, but became unreliable below this point (Fig. S4A-B). To circumvent this, we devised an alternative method for situations when as little as one sample is available, as is the case in clinical settings. This approach relies on kNN-based classification, making it possible to infer SRS/SRSq for each sample independently while retaining cross-technology applicability (Fig. S4C). Although predictive accuracy was reduced for this approach, we observed overall agreement between predictions derived from both methods (Fig. S4D-E). In particular, samples at the extremes of the SRS continuum were reliably identified by both algorithms. Therefore, we included this approach as a secondary function in SepstratifieR. For clarity, we use ‘SepstratfieR predictions’ to refer exclusively to results from random forest models throughout this study.

We applied SepstratifieR to two additional sepsis cohorts (Table S1). Analysis of data from (21) revealed a clear separation between patients and controls, with patients with sepsis segregating into survivor and non-survivor groups (Fig. S5A). These groups matched SepstratifieR’s predictions, with 77% of SRS2 patients surviving, compared to 42% of SRS1 patients. Accordingly, 82% of the SRS3 group were healthy volunteers (Fig. S5B). SRSq was also significantly associated with illness severity and mortality (Fig. S5C; P < 2.2e-16), and SRSq-associated genes observed in GAinS were recapitulated in this cohort (Fig. S5D-F). In particular, high SRSq associated with upregulation of innate immune pathways and downregulation of T cell pathways (Fig. S5G), which was supported by a correlation between SRSq and neutrophil proportions (Fig. S5H). An important feature of this cohort was the availability of temporal information, which enabled us to study changes in SRSq. Whereas SRSq remained constant in healthy individuals, it decreased over time in sepsis (Fig. S5I), particularly so in survivors (p value = 0.0032). Thus, monitoring temporal changes in SRSq could help distinguish patient trajectories.

We then applied SepstratifieR to the Molecular Diagnosis and Risk of Sepsis (MARS) study (7). Four patient clusters have been previously described in this cohort, of which patients in the Mars1 group exhibited higher mortality (7). Mars1 patients separated along the first principal component, whereas SRS1 and 2 groups separated along the second component (Fig. S6A). We observed an overlap between SRS2 and Mars3, as well as an enrichment of Mars2 patients within SRS1. In contrast, 84% of the SRS3 group consisted of healthy volunteers (Fig. S6B). The Mars1 endotype did not correspond to any SRS group, and is thus likely an orthogonal axis of variation. Gene expression differences were highly correlated between studies (Pearson correlation = 0.83), with a similar set of differentially active pathways (Fig. S6C-D). At the clinical level, we did not observe any mortality differences between SRS groups (Fig. S6E). Although this was surprising, 28-day mortality is not always the most relevant outcome measure in critical illness, as it is influenced by factors unrelated to acute illness (for example comorbidities and healthcare settings), and fails to measure quality of life variables in each patient (22). The duration and severity of organ dysfunction are more informative in this regard, and SRS robustly separated MARS patients by organ dysfunction risk, with SRS1 patients characterized by elevated rates of shock (Fig. S6F), higher frequency of acute kidney injury (Fig. S6G) and increased cardiovascular instability (Fig. S6H). Moreover, SRSq also correlated with SOFA (Fig. S6I). These observations suggest that SRS is associated with increased risk of organ dysfunction in the MARS cohort.

Last, we asked why SRS failed to predict mortality in this cohort. We identified two potential causes. First, this could be due to the Marsl signal, which is independent of SRS. We reasoned that combining both signatures could yield better outcome predictions. Secondly, unmeasured confounders could be disrupting the link between SRSq and death. To test the first possibility, we stratified patients by both MARS and SRS and estimated 28-day survival for cross-subphenotype combinations. We confirmed a significantly lower survival in the Mars1 group, and observed that Mars1 patients assigned to SRS1 showed even poorer outcomes (Fig. S6J). This highlights the value of combining different subphenotyping systems. To test the second possibility, we used mediation analysis (Fig. S6K). Although the overall effect of SRSq on death was not significant in this cohort, we observed significant mediation of SRSq on death via shock and organ failure (Fig. S6L; mediation P = 0.002 and 0.024 for shock and SOFA, respectively). This suggests that an increase in SRSq leads to higher probabilities of shock and organ failure, which in turn increase mortality, but that unobserved variables might counterbalance this effect. Thus, SepstratifieR separates patients with sepsis by molecular profile and risk of organ dysfunction, although this is not invariably associated with early mortality.

### Application of SepstratifieR in pediatric sepsis

**Fig. 5:**
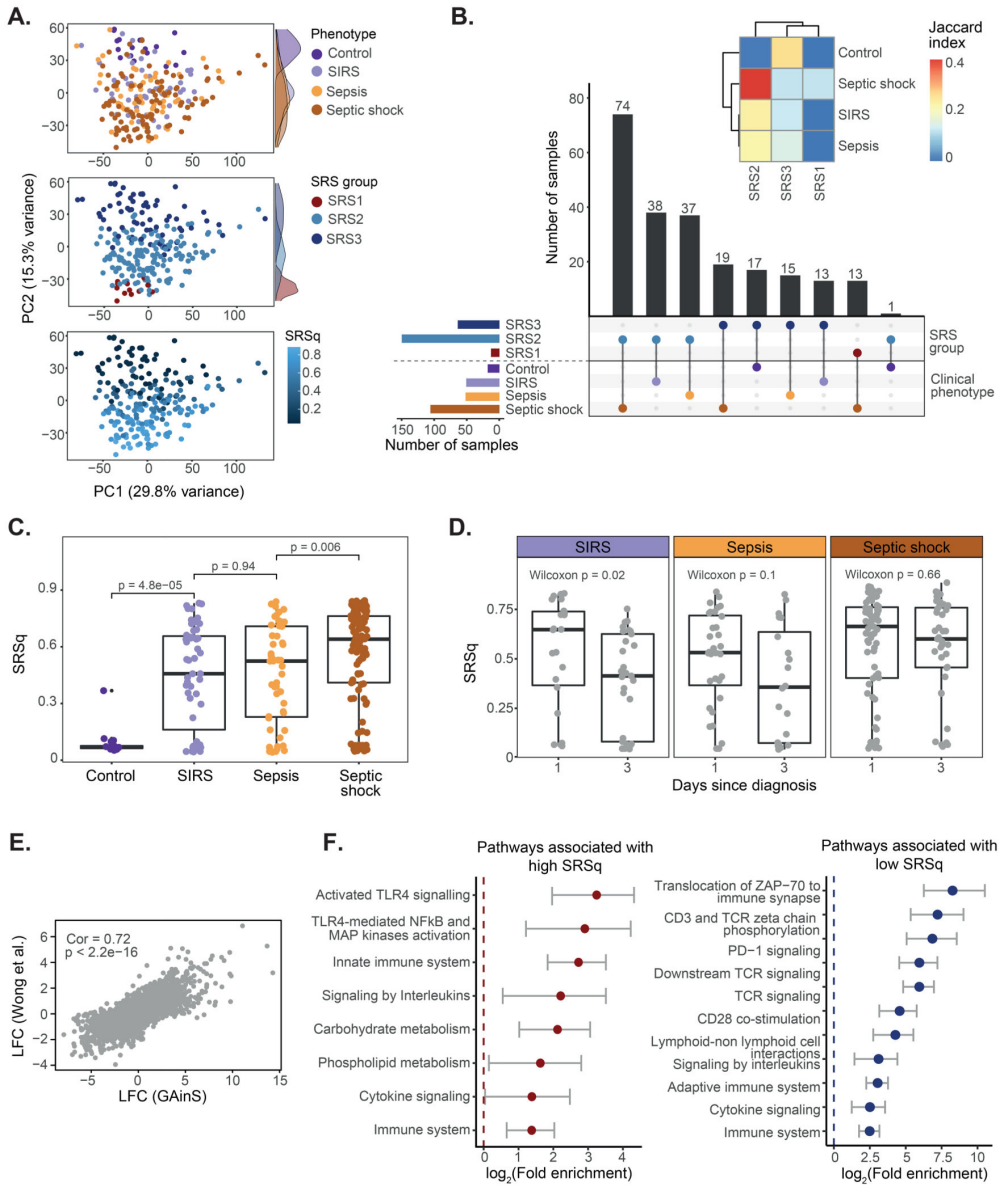
Stratification of patients with pediatric sepsis by SRSq. (**A**) PCA plots based on whole blood transcriptomes. Samples are colored by illness severity (top), SRS (middle), and SRSq (bottom). (**B**) UpSet plot showing the agreement between SRS predictions and disease severity. Bar colors indicate SRS groups (top) and clinical phenotypes (bottom). The heatmap (top) quantifies the extent of this agreement (Jaccard indices). (**C**) SRSq distribution by clinical phenotype; p = Wilcoxon test p value. (**D**) SRSq distribution by time point and clinical phenotype. p = Wilcoxon test p value. (**E**) Correlation between SRSq-associated gene expression changes in adult (GAinS) and pediatric sepsis. Cor = Pearson correlation; p = correlation p value. (**F**) Immune-relevant pathways positively (left) or negatively (right) enriched in SRSq-associated genes.

Several subphenotypes have been described in pediatric sepsis (11, 13). However, the shared and specific features of pediatric vs adult sepsis pathogenesis remain to be fully elucidated. To assess if our models are applicable to pediatric populations, we re-analyzed a cohort of pediatric patients with systemic inflammatory response syndrome (SIRS) (n=23), sepsis (n=38), or septic shock (n=73) (11) (Table S1). Transcriptomic variation in this cohort correlated with clinical severity, with the second principal component separating patients into a progression from health to SIRS, sepsis, and septic shock (Fig. 5A). SRS predictions also associated with PC2, suggesting a correspondence between SRS and illness severity. Indeed, 94% of controls were assigned to SRS3, whereas the SRS1 group consisted exclusively of patients with septic shock and the SRS2 group comprised a mixture of SIRS, sepsis, and septic shock (Fig. 5B). Separation by severity was also apparent for SRSq (Fig. 5C). Last, in agreement with adult sepsis, we observed a decrease of SRSq over time in SIRS patients (Fig. 5D). This confirms that SRSq captures an acute illness signal.

We next asked whether SRSq-associated transcriptional programs were similar in adult and pediatric sepsis. We observed a significant correlation between SRSq-associated genes in both populations (Fig. 5E; Pearson correlation = 0.72, P < 2.2e-16), which was confirmed by pathway enrichment analysis. High SRSq was associated with lower TCR signaling and CD28 co-stimulation, but higher TLR signaling and innate immune pathways (Fig. 5F). This suggests that, although etiologies and immune responses might differ in pediatric patients, most cellular and molecular alterations are shared with adult sepsis, representing mechanisms conserved throughout the lifespan.

### Application of SepstratifieR in influenza and COVID-19

**Fig. 6:**
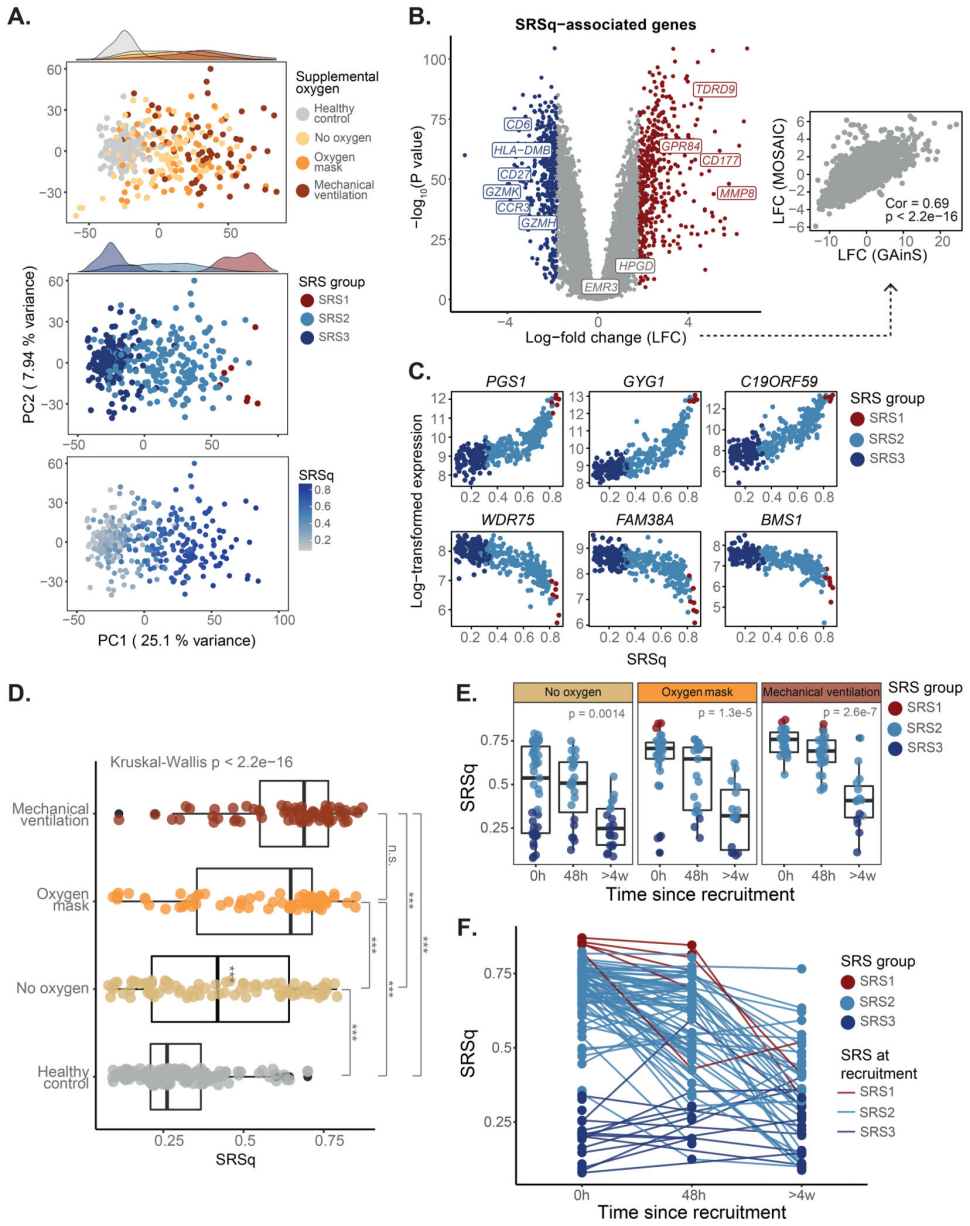
SRSq predicts oxygen requirement and reveals temporal immune dynamics in influenza. (**A**) PCA plots based on whole blood transcriptomes. Samples are colored by oxygen requirement (top), SRS (middle), and SRSq (bottom). (**B**) Volcano plot showing genes differentially expressed along SRSq. Red indicates positive and blue negative associations with SRSq. The scatter plot (right) compares log-fold changes in sepsis (GAinS) and Influenza. Cor = Pearson correlation; p = correlation p value. (**C**) Top genes positively (top) and negatively (bottom) associated with SRSq. Samples are colored by SRS group. (**D**) SRSq stratified by supplemental oxygen requirement; p = Kruskal-Wallis test p value, *** = adjusted Dunn’s post-hoc test p < 0.01. (**E**) SRSq stratified by time since admission and oxygen requirement. Samples are colored by SRS group. p = Kruskal-Wallis test p value. (**F**) Line plot showing changes of SRSq over time. Line colors indicate SRS group assignment at recruitment.

To test whether SepstratifieR is applicable even when patients who do not fulfill sepsis criteria, we deployed it in a cohort of patients hospitalized with influenza (23). This cohort spanned a wide severity spectrum, including patients with and without supplemental oxygen, who would not fulfill the sepsis definition, as well as patients in critical care, who would fulfill Sepsis-3 criteria (24). Exploratory analysis revealed a gradation of illness severities, with patients separating by extent of oxygen supplementation, which was well captured by SRSq (Fig. 6A). At the molecular level, we confirmed increased expression of innate immunity genes proportionally to SRSq (Fig. 6B), with a significant correlation of effect sizes between sepsis and influenza (Pearson correlation = 0.69, P < 2.2e-16; Fig. 6B-C). We next tested the association between SRSq and illness severity. We found an association between SRSq and the extent of oxygen supplementation, with patients on mechanical ventilation showing a higher SRSq than patients without supplemental oxygen (Fig. 6D). In addition, we observed a decrease of SRSq over time, with most patients displaying SRSq values equivalent to healthy volunteers after 4 weeks (Fig. 6E). Whereas patients with high SRSq upon admission (> 0.4) showed variable rates of SRSq decrease, patients with low initial SRSq (< 0.4) showed no changes over time (Fig. 6F). These observations demonstrate that SepstratifieR is applicable to influenza, even when patients do not fulfill sepsis criteria.

**Fig. 7:**
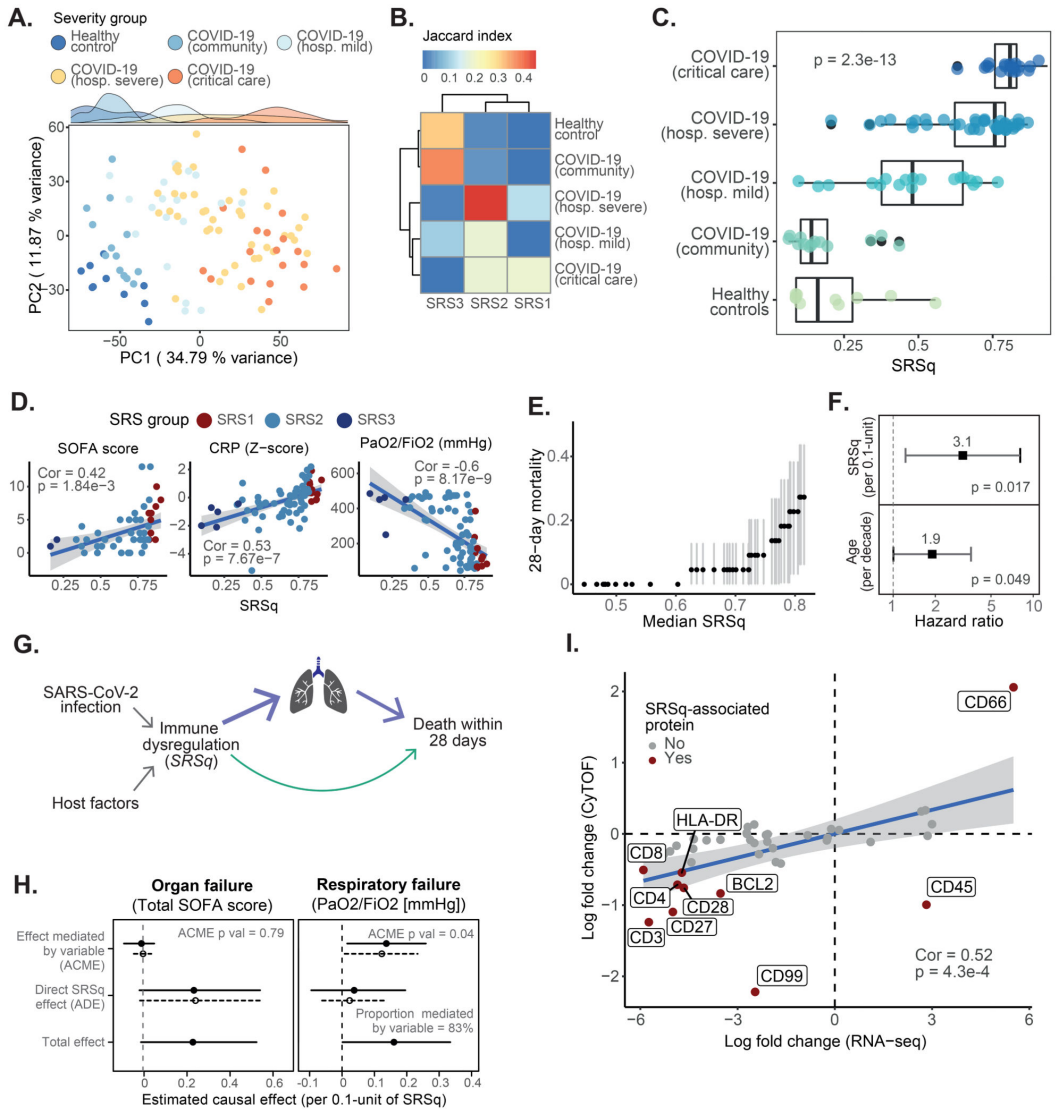
SRSq predicts severity of illness and pinpoints mediators of COVID-19 mortality. (**A**) PCA based on whole blood transcriptomes. Samples are colored by clinical severity. (**B**) Heatmap showing the overlap (as indicated by Jaccard index) between SRS and clinical severity groups. (**C**) SRSq stratified by clinical severity. p = Kruskal-Wallis test p value. (**D**) Association between SRSq and clinical variables. Samples are colored by SRS group. Lines indicate best linear fits. Cor = Pearson correlation; p = correlation p value. (**E**) Association between SRSq and mortality. (**F**) Estimated hazard ratios and 95% confidence intervals. (**G**) Causal model used for mediation analysis. Arrows represent causal directions. (**H**) Results from mediation analysis, with SOFA (left) and P/F ratios (right) as mediators. Lines indicate 95% confidence intervals. Solid and dotted lines represent estimates for the treatment (high SRSq) and control (low SRSq) conditions. ACME = Average Causal Mediation Effect; ADE = Average Direct Effect; p = mediation p value. (**I**) Correlation between SRSq-associated mRNA (x axis) and protein (y axis) changes. Dark red indicates the protein is significantly associated with SRSq. Cor = Pearson correlation; p = correlation p value.

We also applied SepstratifieR to two COVID-19 cohorts: the COVID-19 Multi-Omic Blood Atlas (COMBAT) (20) and the Deutsche COVID-19 Omics Initiative (DeCOI; Table S1) (25). Patients in these cohorts also spanned a wide range of severities, with a proportion presenting with mild disease, others requiring hospitalization, and a subset being admitted to intensive care, many of whom would fulfill sepsis criteria. In both cohorts, whole-transcriptome analysis separated patients and controls, as well as illness severities (Fig. 7A and Fig. S7A). SRS predictions matched with severity groups. In particular, 90% of healthy volunteers in DeCOI were assigned to SRS3, whereas 80% of COVID-19 patients were classified as either SRS1 or SRS2 (Fig. S7B). In COMBAT, the SRS3 group contained a mixture of healthy volunteers and community COVID-19 cases, who were never hospitalized. In contrast, SRS2 and SRS1 were enriched in patients with severe illness and in critical care, respectively (Fig. 7B). SRSq also increased proportionally to illness severity in both cohorts (Fig. 7C and Fig. S7C).

We next compared SRSq-associated pathways between sepsis and COVID-19. We observed a similar set of SRSq-associated genes in both conditions (COVID-19 in DeCOI (Fig. S7D-E), and COVID-19 and sepsis in COMBAT (Fig. S8A-B)). Higher SRSq scores in COVID-19 were associated with downregulation of antigen presentation and T cell pathways, as well as upregulation of TLR signaling, IL-1 signaling, and glycolysis (Fig. S7F and Fig.S8C). Furthermore, SRSq positively correlated with neutrophil counts and negatively correlated with lymphocyte counts (Fig. S7G and Fig. S8D). With regard to clinical measures, SRSq was associated with C reactive protein (CRP), respiratory function (P/F ratios), and SOFA, as well as with pneumonia indexes estimated by DeCOI investigators (Fig. 7D and Fig. S7H). To assess if this resulted in differential outcomes, we evaluated the relationship between SRSq and 28-day mortality in COMBAT. Whereas all participants with SRSq < 0.6 survived, we observed a sharp increase in mortality in patients with SRSq > 0.6 (Fig. 7E). This association was significant (HR = 3.1 per 0.1-unit increase in SRSq, log-rank P = 0.017), even when accounting for age (Fig. 7F). We next asked which factors are involved in death due to COVID-19 using mediation analysis (Fig. 7G) and found no evidence of SOFA scores mediating the effect of SRSq on death. Instead, this effect was explained by respiratory function alone (Fig. 7H). This suggests marked differences between the mechanisms of death in sepsis and COVID-19. In particular, respiratory failure plays a more prominent role in SARS-CoV-2 infection, presumably because patients span a wider severity range and inflammatory response is concentrated in the lungs.

Last, we asked whether SRS was detectable at the protein level. We leveraged leukocyte mass cytometry measurements acquired within COMBAT (20) to match 41 proteins to their corresponding mRNA measurements (from RNA-seq). Having confirmed that both layers of information were correlated (Fig. S8E-G), we explored the relationship between SRS and protein profiles. Exploratory analysis revealed a segregation of samples by severity along the first protein principal component (Fig. S8H), which agreed with SRS labels derived from RNA-seq. The proteins associated with SRSq included CD66 (positively associated), as well as CD3 and CD99 (negatively associated) (Fig. S8I), among others. The direction of these effects agreed between mRNA and protein for 9 out of 10 genes (Fig. 7I). Therefore, SRS is detectable at the protein level in whole blood leukocytes from COVID-19 patients, indicating it might be possible to design cytometry-based assays for SRS/SRSq estimation.

In summary, SRSq is a quantitative score reflective of immune dysfunction and applicable across infections. Elevated SRSq indicates decreased lymphocyte function and antigen presentation, increased neutrophil counts and TLR signaling, more severe illness, and higher risk of poor outcomes. This is explained by alterations in coagulation and blood pressure in sepsis, but by respiratory failure in COVID-19. These factors, possibly in combination with differential responses to immunomodulatory therapy, influence mortality.

## Discussion

We described SepstratifieR, a collection of models for stratification of patients with acute infection which are based on the SRS groups previously described by our group (5). Our study addresses long-standing challenges. First, it furthers our ability to identify subphenotypes and specific endotypes at point-of-care by providing a framework which can be used with rapid turnaround methods (qRT-PCR), as well as full-transcriptome technologies. Second, it models patient disease as a continuum, extending stratification to a range of presentations, independently of whether patients fulfill sepsis criteria. Last, it introduces a quantitative score reflective of immune dysregulation and illness severity, enabling future personalized therapeutic decision making and estimation.

Stratification of sepsis patients has been explored extensively using gene expression (5, 7, 11), clinical variables (10), or circulating biomarkers (13). Our observations align well with these studies, confirming the existence of subgroups of patients with different molecular characteristics. Moreover, SepstratifieR allows clinically relevant stratification in both adult and pediatric populations, revealing overlapping biology between both groups. Previous work has reported pediatric sepsis patient groups associated with pathogen burden (13). These show some correlation but are distinct from SRS (26). Here we show that our signature successfully segregates pediatric patients by illness severity, identifying similar biological pathways to those in adult sepsis, indicating relevance in this population. SepstratifieR also performed well across patients who had different illness severities following viral infection. This indicates that, although both shared and specific disease mechanisms are recognized for influenza and COVID-19 compared with all-cause sepsis (20, 27), these patients are amenable to stratification using SRSq.

By modeling disease as a continuum, we have further supported the concept that patients with sepsis are at the extreme part of a spectrum of variable immune dysregulation, and that our approach enables estimation of immune state and risk regardless of whether patients fulfill the Sepsis-3 definition. Modeling immune risk quantitatively also enabled us to measure immune changes over time. We previously reported that SRS are dynamic, with some patients transitioning between groups during their stay in hospital (6). Here, we showed that SRSq decreases over time along recovery, with larger decreases associated with better outcomes. SRSq therefore represents an important step forward by providing a method to monitor patient progress throughout the course of illness.

SepstratifieR could also inform clinical trial design. Previous trials in sepsis have often failed due to patient heterogeneity (28), with the same treatment potentially harming and benefiting different subgroups of patients (10, 14, 28). SepstratifieR could enable “predictive enrichment” of clinical trial populations for particular immune profiles, as well as “prognostic enrichment” (enrolling only patients at high risk), thus increasing chances of success. Systems biology approaches have also shown that, given the complexity of interactions within the immune system, perturbing immune mediators often results in unpredictable effects due to the emergent properties of immunological networks. Thus, depending on their initial conditions patients could show opposite responses to the same intervention (29, 30), and some form of patient pre-selection is therefore required when trialing therapies. SRSq provides an ideal system for selection and monitoring of patients in such trial designs, as it captures substantial information in a single, quantitative variable.

Nonetheless, our study has some limitations. First, SepstratifieR relies on bulk gene expression, and cannot establish which cellular alterations cause immune dysfunction. Combining SepstratifieR with single-cell technologies is a promising research avenue, as evidenced by our recent study describing differences in granulopoiesis between SRS groups (31). Second, SRSq does not capture the full heterogeneity of sepsis, and orthogonal axes of variation could be lost if focusing exclusively on SRSq. More complete knowledge of the nature and breadth of subphenotypes informative of immune dysregulation, which would likely be used in combination, could circumvent this limitation. Third, our results suggest that SRSq is not invariably associated with early mortality. Although this may be due to unmeasured confounders, it is currently difficult to assess this due to the observational nature of our cohorts. In the future, it will be important to investigate the causes of this discrepancy using prospective cohorts and more comprehensive clinical trial data. Last, our mediation effects are difficult to interpret without further validation, as they rely on the assumption that immune dysfunction increases mortality via its effect on organ failure. Although this agrees with our understanding of sepsis pathophysiology (2, 32), the transcriptional signal captured by SRSq could itself be influenced by shock and organ failure. Prospective studies with dense temporal sampling, in combination with disease models amenable to perturbation, could help address this question.

In conclusion, SepstratifieR enables stratification of patients with acute infection and can model their responses as a continuum. In combination with clinical biomarkers, SepstratifieR could improve risk estimation of immune dysfunction and clinical outcomes, as well as inform clinical trial design, bringing us closer to precision medicine for severe infection.

## Materials And Methods

### Study design and participants

#### The UK Genomics Advances in Sepsis (GAinS) cohort

The UK Genomics Advances in Sepsis (GAinS) study (NCT00121196) aimed to understand the basis of individual variation in the sepsis response for patients admitted to intensive care by combining detailed clinical phenotyping with molecular, genetic and functional genomic profiling of peripheral blood. Adult patients (≥18 yo) admitted into intensive care with sepsis due to community-acquired pneumonia (CAP, n=688) or fecal peritonitis (FP, n=358) were recruited from 34 UK intensive care units (ICUs) between 16/11/2005 and 30/05/2018. Diagnoses were based on ACCP/SCCM guidelines.

Ethics approval was granted nationally and locally, with informed consent obtained from all patients or their legal representative at the beginning of the study. This research was conducted under Research Ethics Committee approvals 05/MRE00/38, 08/H0505/78, and 06/Q1605/55.

### Procedures

#### Sample collection

Whole blood (~10 mL) was obtained from patients on the first, third, and/or fifth day following ICU admission. Leukocyte isolation was performed at the bedside using the LeukoLOCK system (Thermo Scientific), with RNA extracted using the Total RNA Isolation Protocol (Ambion).

#### Microarray profiling

Blood samples from patients within the GAinS study (Table S1) were processed in four batches, of which three have been previously published. The first batch contained the first available sample from 265 patients with sepsis due to CAP (5); the second batch comprised the first available sample from 106 patients with sepsis due to CAP (50% of whom were non-survivors), and 53 patients with sepsis due to FP (5, 6); the third batch comprised 127 samples from 73 patients with sepsis due to CAP and 94 samples from 64 patients with sepsis due to FP (6); and the fourth batch comprised 24 patients with sepsis due to CAP and 24 patients with sepsis due to FP who had RNA collected at three time points and for whom no gene expression data had previously been generated. Batch 3 included 56 replicate samples from batch 1, which were removed prior to data combination. Outlying samples within each batch were identified and sample mix-ups were resolved using genotype information, resulting in a final set of 676 samples.

The four data sets were combined and normalized together using variance stabilization and normalization with the *vsn* package. Probes with a detection p-value < 0.05 in fewer than 5% of samples were removed, and batch effects were corrected using the *sva* package.

#### RNA-sequencing

RNA-seq was performed on 864 samples (667 patients) from the GAinS study, including 134 samples with previous microarray data (Table S1) plus 533 patients who were not included in the microarray cohort. cDNA libraries were prepared using NEB Ultra II Library Prep kits (Illumina) and sequenced in a NovaSeq 6000 (Illumina). Reads were aligned to the reference genome (GRCh38 v99) using STAR and quantified using featureCounts. Counts were normalized and transformed to log-counts per million.

#### qRT-PCR profiling

Seven genes predictive of SRS and two control housekeeping genes (*ACTB* and *TOP1*) were profiled using qRT-PCR in 115 RNA samples (107 patients) from the GAinS study.

#### Public data collection

Publicly available transcriptomics data were collected from three cohorts of healthy, as well as six infectious disease cohorts comprising all-cause sepsis, influenza, and COVID-19 (Table S1) (7, 20, 21, 23, 25, 33–37). For microarrays, probes were quality filtered (detection p value < 0.01 in over 20% of samples), and measurements averaged across all probes uniquely mapping to each gene. For RNA-seq, counts were normalized and log-transformed, with genes quality filtered (>1 CPM in over 10% of samples).

### Statistical analysis

#### Cross-technology data integration

Canonical correlation analysis (CCA) was performed using sparse CCA with the *PMA* package. Gene contributions to the first canonical dimension (CC1) were used to identify candidate genes for an Extended signature predictive of SRS. Genes were included in the signature if they were amongst the top 1% genes with highest contribution to CC1, and were reliably detected across all the healthy volunteer cohorts used as comparator groups throughout this study (Table S1). This resulted in 12 new genes, which were combined with the Davenport signature to yield an extended 19-gene signature.

After CCA, datasets in the GAinS study were integrated with healthy volunteer data from three cohorts (Table S1) based on either the 7 or the 19-gene signature. Technical differences between studies were removed using mutual nearest neighbors (15) with the *batchelor* package. Batches were defined based on profiling technology (Illumina HumanHT-12 arrays, RNA-seq, or qPCR). This resulted in two cross-technology data sets: the Davenport and the Extended reference sets.

#### Definition of a quantitative sepsis response score

Diffusion maps were constructed based on genes in the Davenport and Extended signatures using the *destiny* package. The first diffusion component (DC1) was used to derive a quantitative sepsis response score (SRSq), defined as the min-max scaled DC1 coordinate:

$$SRSq_{i}=\frac{DC1_{i}-min\left(DC1\right)}{\left(DC1\right)-min\left(DC1\right)}$$

Where **SRSq_i_** and **DC1_i_** represent the quantitative sepsis response signature score and DC1 coordinate for the i-th sample, respectively. Min-max scaling restricts SRSq to the [0,1] range, where values closer to one indicate more severe immune dysfunction. SRSq scores were independent of the technology used for gene expression profiling.

#### Random forest training and evaluation

Random forests were trained using the *randomForest* and *caret* (packages. Five hundred decision trees were built per forest, using either 7 or 19 genes as predictor variables. SRS or SRSq were used as response variables. Performance was evaluated using leave-one-out cross-validation (LOOCV) by estimating Cohen’s Kappa (for SRS) or root-mean-square errors (RMSE; for SRSq).

#### Differential expression analysis

Differential gene expression between SRS and along SRSq was assessed using moderated T-tests with *limma* (38). Genes were deemed differential expressed when: 1) |fold-change| > 1.5 between SRS groups at a false discovery rate (FDR) < 0.05; or 2) |fold-change| > 3.5 per unit increase in SRSq at an FDR of 0.05 (equivalent to a one-fold increase in expression per 0.3 SRSq units).

#### Pathway enrichment analysis

Pathway enrichment was assessed using *XGR* and pathways listed in REACTOME (39). Pathways were deemed significantly enriched at FDR < 0.05.

#### Clinical data collection

GAinS clinical information was collected by local investigators using electronic case report forms (Table S2). Data was quality filtered and assembled into a database for ease of access.

#### Integration of clinical variables with SRS/SRSq

Associations between SRS and clinical variables were tested using Kruskal-Wallis one-way analysis of variance (numeric variables) or Mood’s median test (ordinal variables). Associations between SRSq and clinical variables were assessed using correlation tests (numeric variables) or proportional odds logistic regression (ordinal variables).

#### Survival analysis

Mortality and time to death were censored (or capped) at 28 days after ICU admission. Kaplan-Meier curves were then built, with time to event modeled as a function of SRS (as measured at the latest time point available per patient). Visualization was performed using *survminer*, with significance estimated by log-rank tests.

To test for associations between SRSq and survival, samples were sorted by increasing SRSq (at the latest time point available) and a sliding window containing 35% of samples was used to estimate 28-day survival. Windows were slid one sample at a time until reaching the sample with highest SRSq. Associations were also tested using Cox Proportional Hazard models, where hazard ratios (relative to 0.1-unit increases in SRSq) were modeled as an exponential function of SRSq, adjusting for age and source of sepsis (CAP or FP). Significance was assessed by log-rank tests.

#### Analysis of temporal SRSq dynamics

A subset of 177 patients from the GAinS RNA-seq cohort, profiled at up to three time points following ICU admission (1st, 3rd, or 5th day in ICU), were used for this analysis. Temporal changes in SRSq were defined as log_2_-fold changes (LFC) between the first and last time point. To account for time intervals between samples, LFCs were normalized to interval duration. This resulted in average daily LFCs, which represents the average change in SRSq exhibited per day. Patients were sorted increasingly by average daily LFC in SRSq for survival analysis.

To assess survival, patients were divided into quartiles based on their temporal change in SRSq and Kaplan-Meier curves were built, with significance determined using log-rank tests. Sorted patients were also used to define a sliding window containing 35% of samples. This window was used to estimate 28-day mortality, with sliding performed one sample at a time until reaching the patient at the top of the list.

## Supplementary Material

Supplementary Materials

## Data Availability

Codes are available at https://doi.org/10.5281/zenodo.7079357. The SepstratifieR package can be installed directly from GitHub and is available at Zenodo (https://doi.org/10.5281/zenodo.7079384). Gene expression data for GAinS study samples are publicly available in ArrayExpress (E-MTAB-4421, E-MTAB-4451, E-MTAB-5273, and E-MTAB-5274). Accession numbers for all public datasets used are listed in Table S1. This research was funded in whole or in part by The Wellcome Trust [Grant numbers 204969/Z/16/Z, 206194, 108413/A/15/D, 090532/Z/09/Z and 203141/Z/16/Z], a cOAlition S organization. The author will make the Author Accepted Manuscript (AAM) version available under a CC BY public copyright license.

## One Sentence Summary

Machine learning stratifies patients with infection for personalized immune response profiling as well as clinical severity and outcome prediction.

## Editor’s summary: Stratifying sepsis

Predictors of severe infection could help physicians manage clinical care. Cano-Gamez *et al*. present a RNAseq-based gene expression signature derived from patients with sepsis that generally captured patient prognosis with high sensitivity. Biologically, this signature corresponded to immune dysfunction. A machine learning framework based on the gene signature correctly stratified pediatric and adult patients with bacterial or viral sepsis, as well as patients with infection who did not meet sepsis criteria, including H1N1 influenza and COVID-19. This study may thus advance our ability to predict individual patient outcomes in sepsis.
