# Supplementary material for: An immune dysfunction score for stratification of patients with acute infection based on whole blood gene expression
Source: Sci Transl Med. Author manuscript; Available in PMC 2022 Nov 16. (PMC7613832; doi:10.1126/scitranslmed.abq4433)
Supplement: Supplementary Materials [file EMS156835-supplement-Supplementary_Materials.pdf]

SUPPLEMENTARY MATERIALS

SUPPLEMENTARY METHODS

Procedures

qRT-PCR profiling

Quantitative RT-PCR was used to quantify the seven genes in the Davenport SRS signature *(5, 6)*, plus two control housekeeping genes (*ACTB* and *TOP1*) in 115 RNA samples (corresponding to 107 patients) from the GAinS study.

Primers were designed using NCBI's Primer-BLAST, ensuring that primers were exon-exon spanning, and amplicons were 70-250 bp and within 500 bp of a corresponding Illumina microarray probe (**Table S3**). A Basic Local Alignment Search Tool (BLAST) search was performed against Ensembl GRCh37 v97 to ensure absence of homology to other genomic regions (E-value <0.01).

qRT-PCR was conducted on patient samples, two non-targeting controls (NTCs), and a healthy volunteer positive control, with cDNA generated from 500 ng of RNA using the LunaScript RT SuperMix Kit (New England Biolabs). Amplification was performed using a 20 µL qPCR reaction containing the following: 2 µL cDNA (diluted at a 1 in 5 ratio), 10 µL iQ SYBR Green Supermix (BioRad), 2 µL primer mix (1 µL of forward and 1 µL reverse primers), and 6 µL nuclease-free water. This reaction was carried out using the following cycle parameters: 1) 94°C for 2 min; 2) 40 cycles of 94°C for 30s, 56.5°C for 30s, and 72°C for 45s; and 3) 72°C for 5 min.

Raw data were processed with CFX Manager (BioRad), and samples with Cq > 35 were removed. Cq values for each gene were then averaged across all technical replicates available for each patient. Next, Cq values were normalized to the control genes by subtracting the geometric mean of housekeeping genes from each gene in the SRS signature, as follows:

$$Cq_i\, norm = Cq_i - \sqrt{Cq_{ACTB} Cq_{TOP1}}$$

Where *i* represents the gene in the SRS signature which is being analyzed. Last, normalized Cq values were batch corrected using *limma* (v3.44.1) and used for further analyses.

Statistical analyses

Mediation analysis

Mediation analysis was implemented using the *mediation* package, with effect sizes estimated relative to 0.1-unit increases in SRSq. This analysis is best conceptualized using causal diagrams. In the diagram below, X represents an independent variable and Y a dependent variable, with M being a mediator lying in the causal path between X and Y. Arrows indicate the direction of causality:

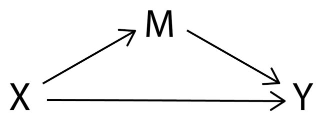

Mediation analysis estimates how much of the effect of **X** on **Y** depends on changes in **M** (the average causal mediation effect, ACME), as well as how much is independent of **M** (the direct causal effect, ADE) *(18)*. To do so, **Y** is modeled as a function of **M** and **X**, with **M** itself being a function of **X**. ACME and ADE are then estimated either exactly or with simulations.

In this study, SOFA, organ function measurements, and the presence/absence of shock were considered potential mediators of SRSq on mortality. Thus, each of them was modeled as a function of SRSq while accounting for covariates (age and source of sepsis). More specifically, numeric variables (e.g. SOFA) were modeled using a standard linear regression:

$$M = \alpha_0 + \alpha_1 SRSq + \sum_{i=1}^{n} \alpha_{i+1} C_i + \varepsilon$$

Where **M** is the mediator variable, $\mathbf{C_i}$ the i-th covariate, and $\boldsymbol{\alpha}$ and $\boldsymbol{\varepsilon}$ the regression coefficients and random error term, respectively.

In contrast, binary variables (e.g. shock) were modeled using logistic regression with a probit model:

$$p(M) = \Phi\left(\alpha_0 + \alpha_1 SRSq + \sum_{i=1}^{n} \alpha_{i+1} C_i\right)$$

Where **p(M)** is the probability of the mediator, $\mathbf{C_i}$ the i-th covariate, $\boldsymbol{\alpha}$ and $\boldsymbol{\varepsilon}$ the regression coefficients and random error term, respectively, and $\boldsymbol{\Phi}$ the cumulative distribution function (CDF) of the normal distribution:

$$\Phi(x) = p(X \le x)$$

Assuming **X** is normally distributed with zero mean and unit standard deviation:

$$X \sim N(0,1)$$

The dependent variable (i.e. mortality) was then modeled as a function of each mediator, one variable at a time, while accounting for SRSq and covariates. This was done using a logistic regression with probit model:

$$p(D) = \Phi\left(\beta_0 + \beta_1 M + \beta_2 SRSq + \sum_{i=1}^{n} \beta_{i+2} C_i\right)$$

Where **p(D)** is the probability of death within 28 days of ICU admission, **M** the mediator of interest, $\mathbf{C_i}$ the i-th covariate, $\boldsymbol{\alpha}$ and $\boldsymbol{\varepsilon}$ the regression coefficients and random error term, respectively, and $\boldsymbol{\Phi}$ the cumulative distribution function (CDF) of the normal distribution.

ACMEs, ADEs, and their 95% confidence intervals were estimated using Monte Carlo simulations, as implemented in the *mediation* package (v4.5.0). Effect sizes were estimated relative to 0.1-unit increases in SRSq.

Mediation effects are best interpreted in terms of counterfactuals *(18)*. In particular, ADEs tell us how much mortality would increase if SRSq were increased by 0.1 units while keeping the mediator constant. In contrast, ACMEs tell us how much mortality would increase if SRSq were held constant, but the mediator was artificially increased as if SRSq had increased by 0.1 units.

The SepstratifieR algorithm

SepstratifieR was developed using R and is publicly available for download and installation from our GitHub repository (https://github.com/jknightlab/SepstratifieR). SepstratifieR contains two independent approaches for patient stratification: 1) prediction based on pre-trained and cross-validated random forest models (recommended mode), and 2) mNN-based prediction (recommended only for situations where samples are profiled individually or sample size drops below 20). The algorithms followed by each of these approaches are detailed below.

A) Random forest-based classification

Through the *stratifyPatients()* function, the SepstratifieR package provides the user with access to a set of validated random forest classification and prediction models to infer SRS and SRSq based on either the Davenport or the Extended gene signature. When this function is called, the following algorithm is followed:

1. The samples of interest (i.e. the user's input) are aligned to the samples in the Davenport of Extended reference set (as specified by the user) so as to remove differences between batches or technologies. This alignment is performed using the mutual nearest neighbors (mNN) algorithm *(15)*, with the number of nearest neighbors specified by the user. This results in a set of batch-aligned expression measurements.
2. The pre-trained random forest models introduced in this study are used to classify samples into SRS groups based on the batch-alignment set of variables.
3. The pre-trained random forest models introduced in this study are used to predict SRSq for each sample.
4. The algorithm returns an object containing raw expression measurements, batch-aligned expression measurements, SRS predictions, SRSq predictions, and information on the parameters used to run the algorithm.

We recommend this algorithm as the default way of assigning SRS and SRSq in new data sets. Further information on this function, as well as documentation of the associated R code, and an associated method to perform sensitivity analysis and determine the ideal number of nearest neighbors are available at https://github.com/jknightlab/SepstratifieR.

B) kNN-based classification

This approach relies on cosine similarities, a measure of the similarity between two data points which does not depend on the magnitude of each variable, but rather on the relationships (i.e. ratios) between different variables (in this case, the 7 or 19 genes in the signature). If we conceptualize each sample as a vector in space (each gene being a dimension), cosine similarity represents the cosine of the angle between two vectors. This angle is independent of vector length, and only depends on the direction in which vectors are pointing. This makes cosine similarities scale-independent and robust to technical differences. A cosine similarity of 1 means two samples are identical (i.e. vectors point in the same direction), a similarity of 0 means they are orthogonal, and a similarity of -1 means they are antiparallel (i.e. vectors point in opposite directions). Thus, cosine similarities are here used to compare a query sample to all samples in the reference sets, thus enabling identification of the top samples most similar to the query patient. Next, these samples are used to infer SRS groups and SRSq using a kNN-based classification system.

This algorithm is available via the *projectPatient()* function in the SepstratifieR package, and consists of the following steps:

1. Cosine similarities are calculated between the query and all the reference samples
2. The k samples with highest cosine similarity to the query (default k = 20) are identified. These are referred to as the k nearest neighbors.
3. An SRS label is assigned to the query sample based on a 'majority vote' system, where each nearest neighbor gets a vote with a weight proportional to its cosine similarity. The query sample is assigned to the SRS category with the highest vote count.
4. An SRSq value is predicted for the query by calculating a weighted average of SRSq across its nearest neighbors. Each neighbor's contribution is weighted by its cosine similarity with the query.

This algorithm follows a 'lazy learning' approach, because it does not rely on trained and validated machine learning models, but rather on distance calculations. The advantage of this is that it obviates the need for batch alignment and is applicable to isolated samples. The drawback is that distance measurements must be calculated every time a sample is classified, making the approach computationally intensive, and that it cannot learn structure in the way a machine learning model does.

We recommend this approach in situations where samples are either profiled individually, one at a time, or in batches with less than 20 samples each.

CyTOF data analysis

Mass cytometry measurements from whole blood leukocytes were from COMBAT *(20)*. Protein abundances were averaged across all cells from each individual to create *pseudobulk* protein profiles. Principal component analysis was used for dimensionality reduction. The agreement between mRNA and protein was assessed using Pearson correlation tests, with Bonferroni correction for multiple testing. Associations between protein abundance and SRSq were tested using linear models with *limma*, with FDR correction for multiple testing. Proteins were deemed SRSq-associated if $\log_2$-fold changes ≥ 0.5 at $FDR < 0.05$.

SUPPLEMENTARY FIGURES

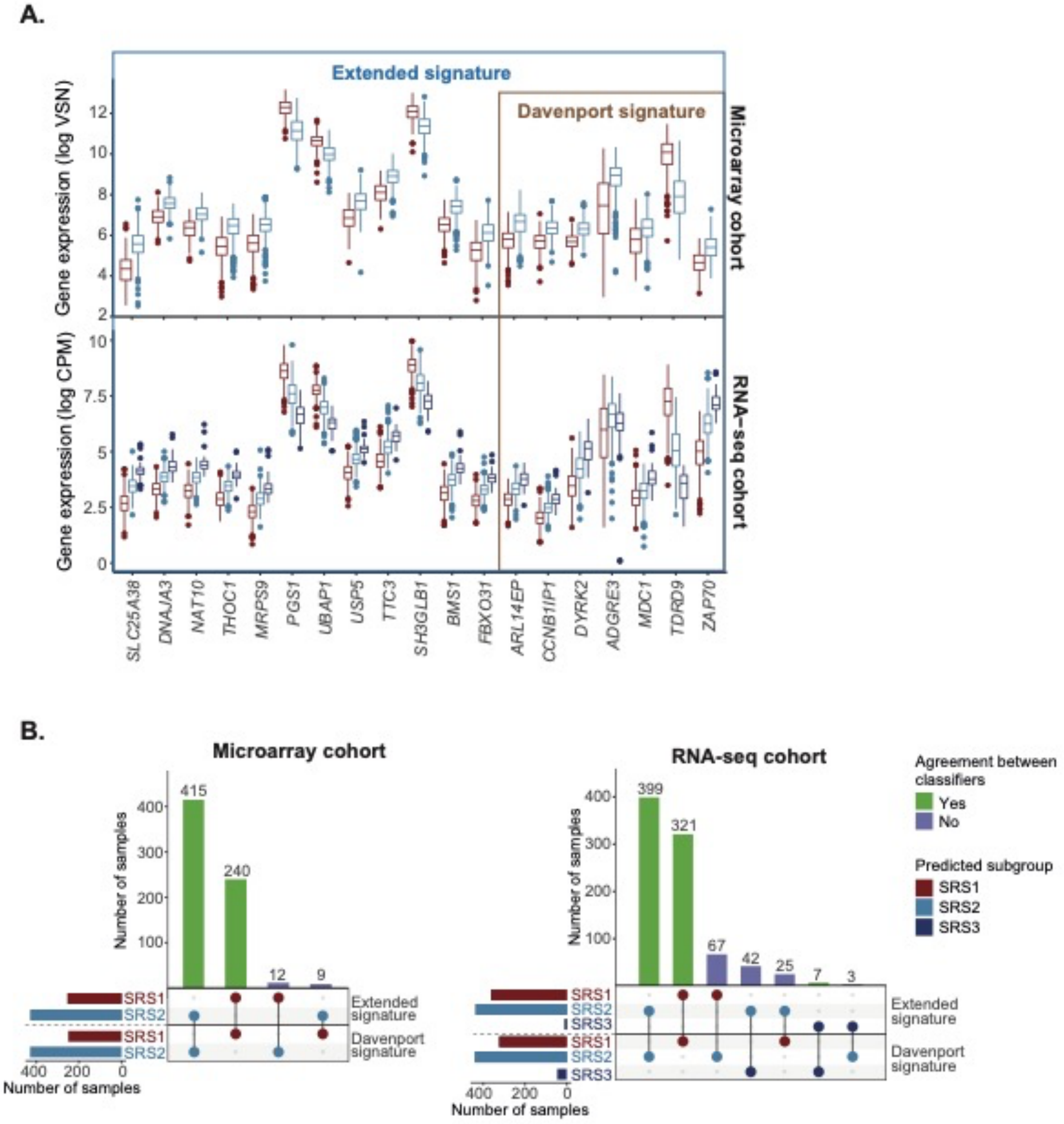

Fig. S1. Two gene signatures for stratification of patients with sepsis. (**A**) Box plots showing the expression level of genes in the Davenport and Extended signatures, as measured using microarrays (top) and RNA-seq (bottom). Samples are stratified by sepsis response signature (SRS) group (red = SRS1, light blue = SRS2, dark blue = SRS3). (**B**) UpSet plot showing the agreement between SRS predictions from the Davenport and Extended signatures in microarray (left) and RNA-seq (right) GAinS samples. Bar heights indicate the number of samples in each set, with bar colors representing SRS groups (horizontal) and cross-signature agreement (vertical).

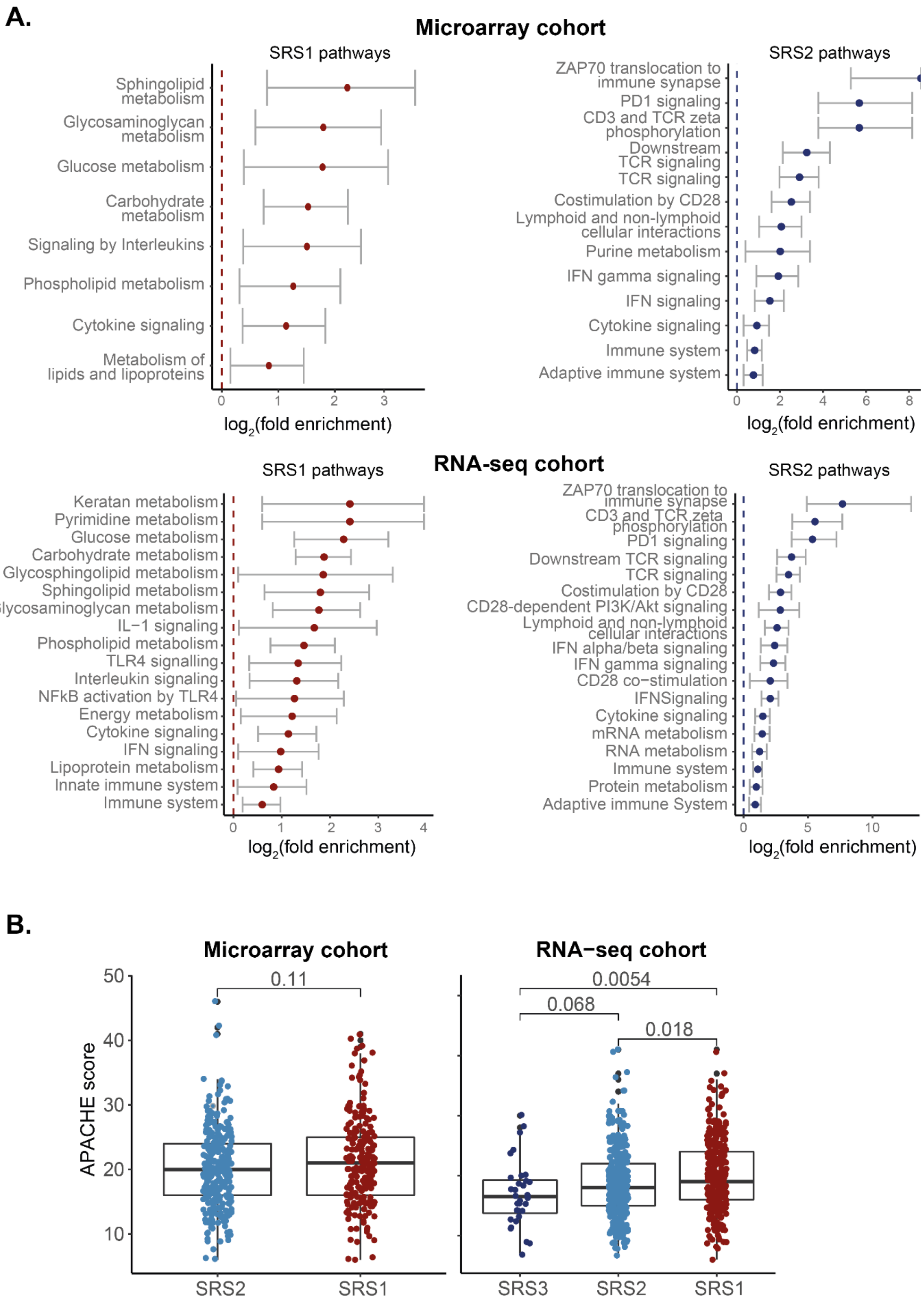

Fig. S2. Features of SRS groups in the GAinS study. (**A**) A subset of immune-relevant pathways significantly enriched (FDR < 0.05) for genes differentially expressed between SRS groups in the GAinS microarray (top) and RNA-seq (bottom) cohorts. Both SRS1 (left) and SRS2-associated (right) pathways are shown. Whiskers indicate 95% confidence intervals. (**B**) Box plots showing APACHE II scores for each SRS group in the GAinS microarray (left) and RNA-seq (right) cohorts. T test (left) and Kruskal-Wallis test (right) p values are shown.

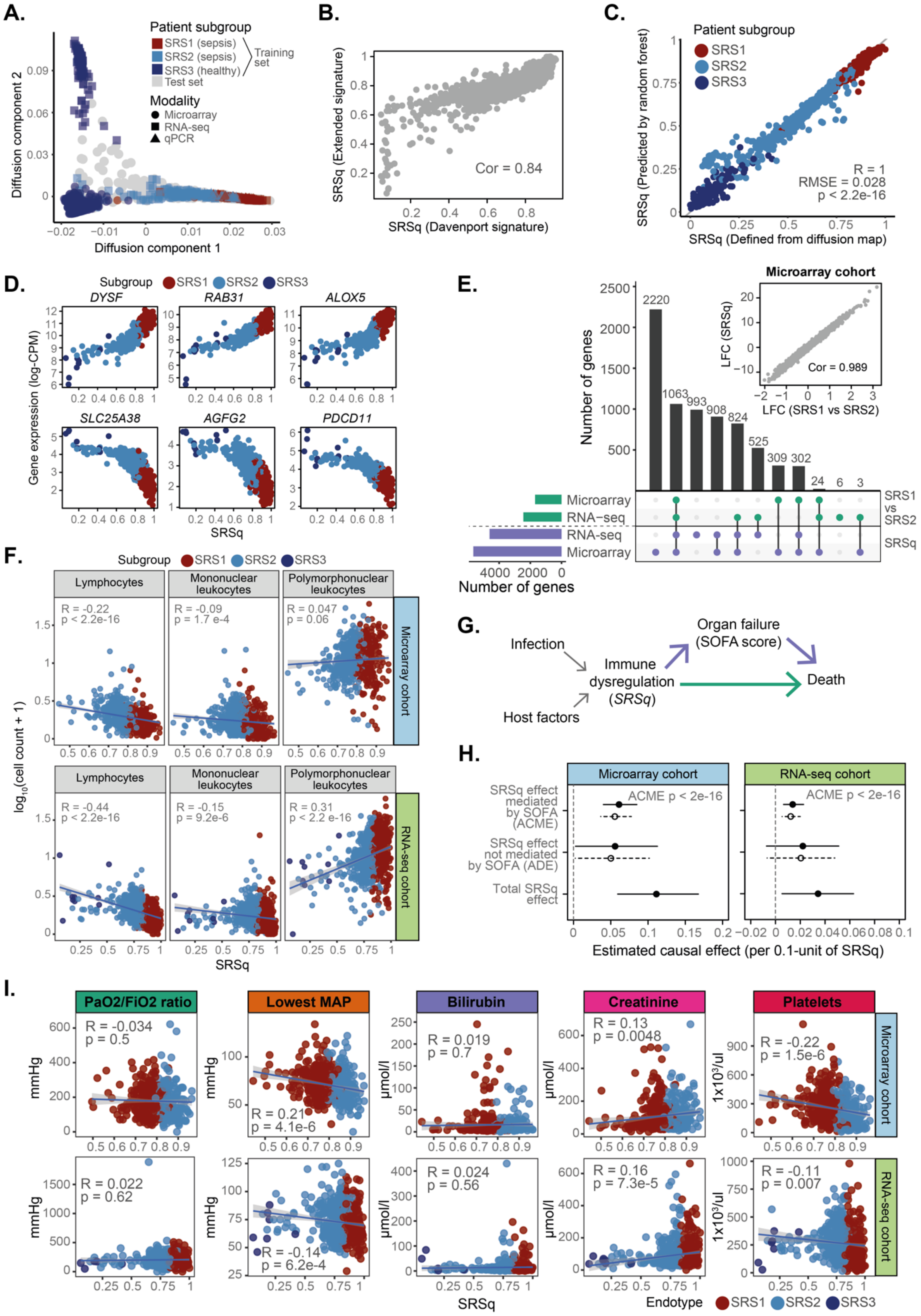

Fig. S3. Clinical characteristics associated with SRSq. (**A**) Diffusion map built using the Davenport signature. Colors indicate SRS groups and shapes profiling platforms. (**B**) Correlation between SRSq predictions from the Davenport and Extended signatures. Cor =

Pearson correlation; P < 2.2e-16. (**C**) Correlation between diffusion map and random forest-derived SRSq. Samples are colored by SRS group. R = Pearson correlation; RMSE = Root Mean Square Error; p = Correlation p value. (**D**) RNA-seq measurements of example genes positively (top) and negatively (bottom) associated with SRSq. Samples are colored by SRS group. (**E**). Comparison between SRSq and SRS-associated differential gene expression. The UpSet plot (bottom) indicates the number of genes deemed differentially expressed by each approach. The scatter plot (bottom) compares log-fold changes between SRS and SRSq. Cor = Pearson correlation coefficient. (**F**) Association between SRSq and cell counts. Samples are colored by SRS group. Lines indicate best linear fits with 95% confidence intervals. R = Pearson correlation; p = correlation p value. (**G**) Causal model assumed for mediation analysis in the GAinS cohort. (**H**) Mediation analysis results in GAinS. Lines indicate 95% confidence intervals. Solid and dotted lines represent estimates for the treatment (high SRSq) and control (low SRSq) conditions. ACME = Average Causal Mediation Effect; ADE = Average Direct Effect; p = mediation p value. (**I**) Correlation between SRSq and clinical variables in GAinS. Samples are colored by SRS group. Lines indicate best linear fits with 95% confidence intervals. R = Pearson correlation; p = correlation p value.

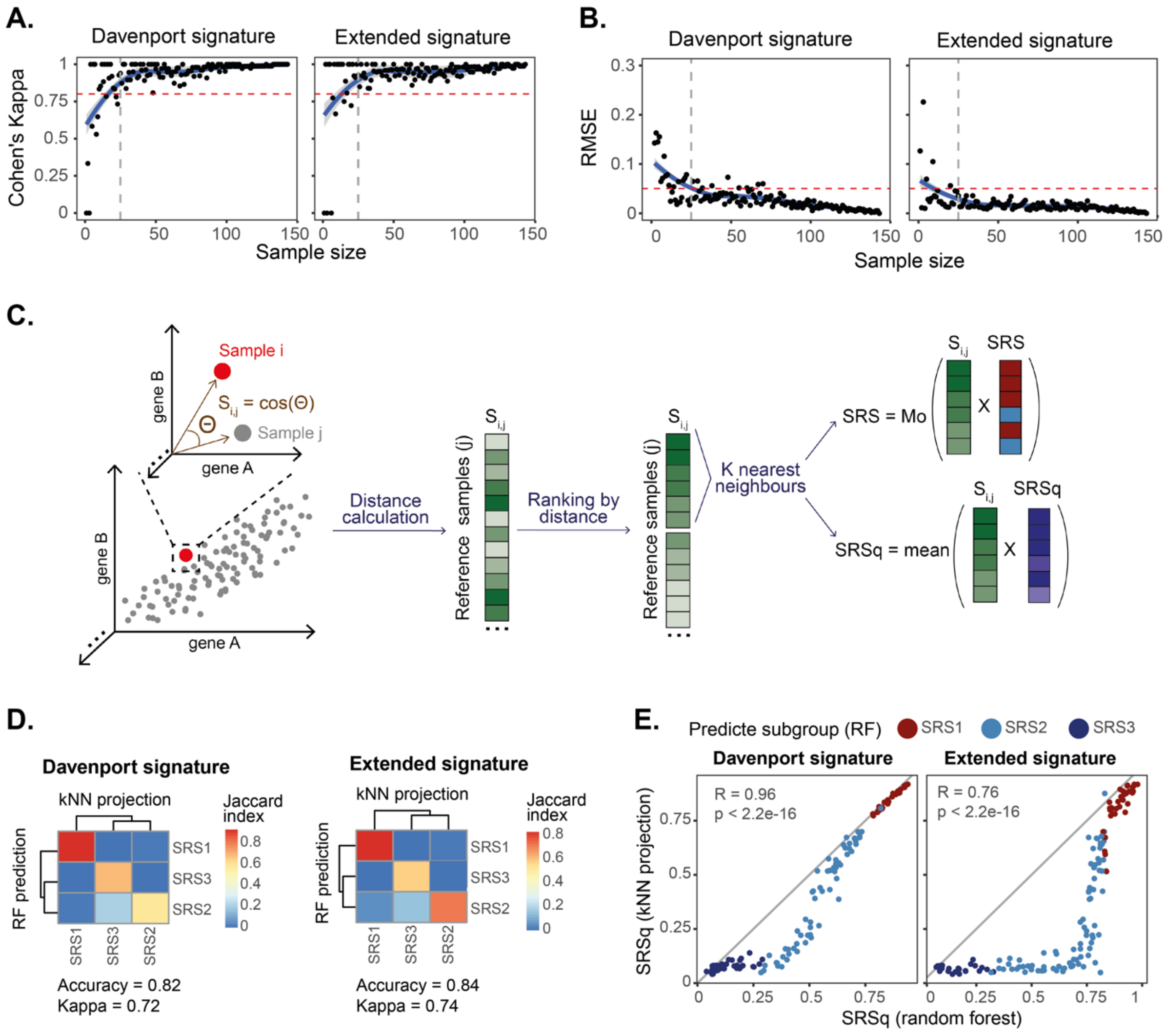

Figure S4. A knn-based 'lazy learning' approach for stratification of isolated samples. (**A**) SRS prediction was repeatedly performed for patients in the COMBAT study by randomly subsampling an increasingly larger number of patients and applying SepstratifieR. These plots

show the classification accuracy (Cohen's Kappa, y axis) achieved for different sample sizes (x axis) using each gene signature. (**B**) SRSq prediction was repeatedly performed in random subsamples of patients. These plots show the prediction accuracy (Root-mean-square error, y axis) achieved for different sample sizes (x axis) using each gene signature. (**C**) Schematic representation of a new approach for stratification of individual samples into SRS groups using kNN-based label projection. The query sample is first compared to all samples in the reference set using cosine similarities. Next, the k most similar samples in the reference (i.e. nearest neighbors) are identified. Last, SRS and SRSq are inferred based on the nearest neighbors. (**D**) Heatmaps showing the extent of overlap (i.e. Jaccard index) between SRS groups defined using random forest models and kNN-based classification based on each gene signature. (**E**) Scatter plots showing the correlation between SRSq predictions obtained using random forest models and kNN-based prediction.

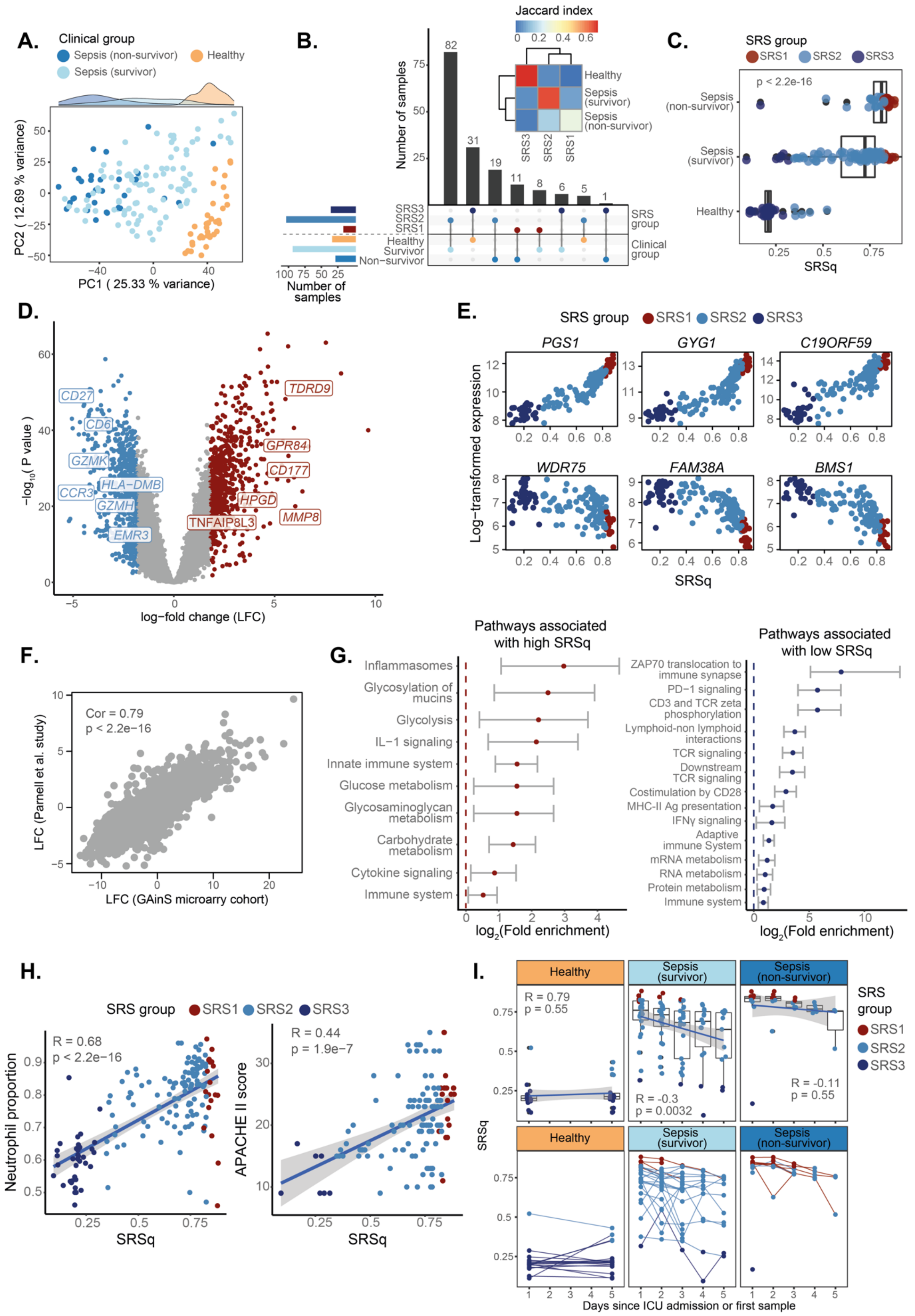

Fig. S5. SRSq validation. (**A**) Whole transcriptome PCA plot. Samples are colored by clinical group. (**B**) UpSet plot (bottom) showing overlaps between SRS and clinical groups. Heatmap

(top) indicating the extent of overlap (i.e. Jaccard index). (**C**) SRSq stratified by clinical group. Samples are colored by SRS group. p = Kruskal-Wallis p value. (**D**) Volcano plot of SRSq-associated genes. Red indicates positive and blue negative associations. (**E**) Example genes positively (top) and negatively (bottom) associated with SRSq. Samples are colored by SRS group. (**F**) Correlation between log-fold changes in GAinS and in data from *(21)*. Cor = Pearson correlation; p = correlation p value. (**G**) Subset of immune-relevant pathways positively (left) or negatively (right) enriched in SRSq-associated genes. (**H**) Association between SRSq and clinical variables. Samples are colored by SRS group. Lines indicate best linear fits and 95% confidence intervals. R = Pearson correlation coefficient; p = correlation p value. (**I**) SRSq stratified by time point. Samples are colored by SRS group, with regression lines representing best linear fits. R = Pearson correlation coefficient, p = correlation p values. Line plots (bottom) connect samples from the same patient, with lines colored by SRS group at the earliest time point.

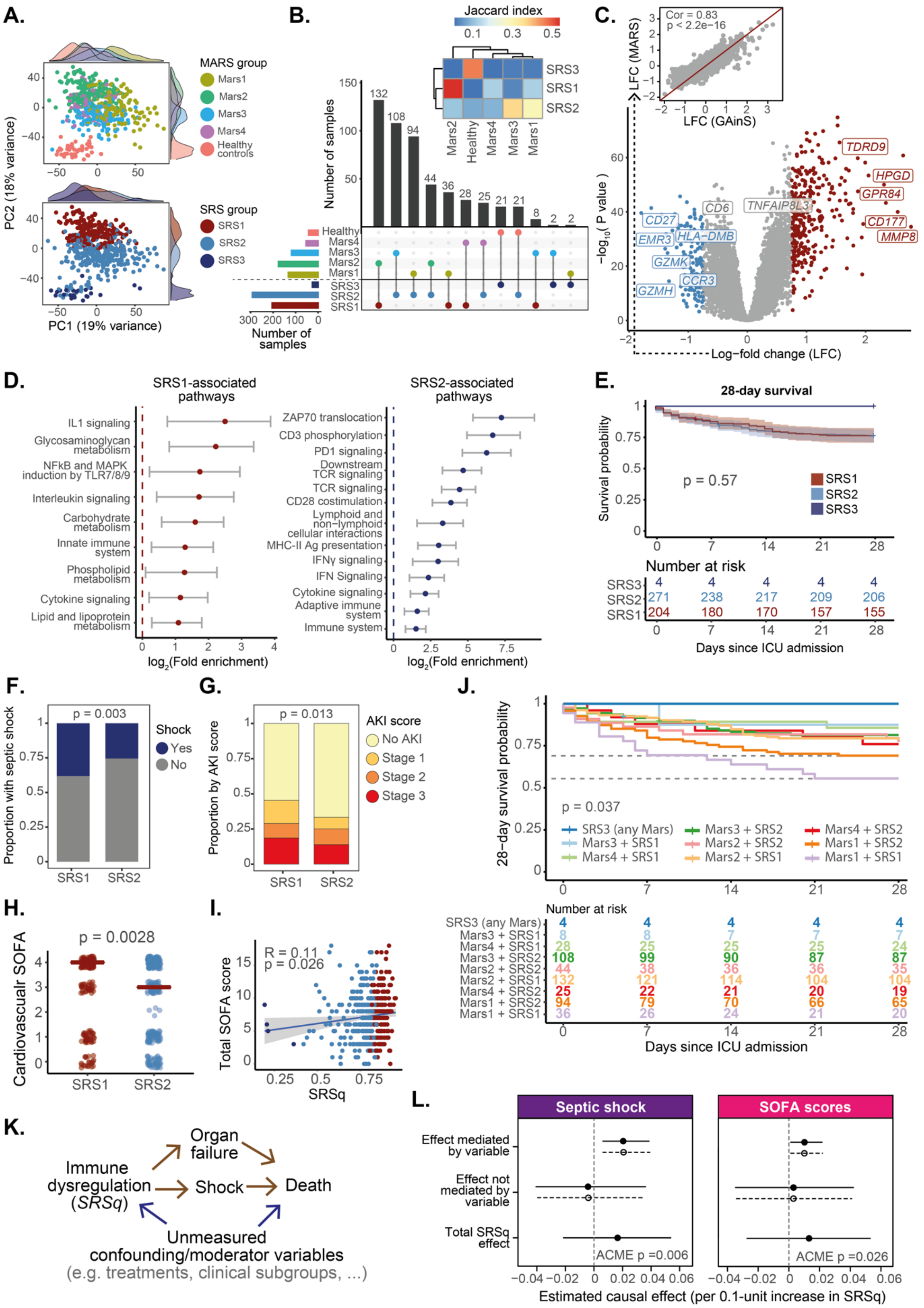

Fig. S6. SRSq validation in the MARS study. (**A**) PCA plot colored by Mars (top) and SRS (bottom) patient grouping. (**B**) UpSet plot showing overlaps between SRS and Mars patient groupings. The heatmap (top) shows a quantitative measure of these overlaps (i.e. Jaccard

indexes). (**C**) SRS1/SRS2 volcano plot. Red indicates upregulation and blue downregulation in SRS1. The scatter plot (top) compares effect sizes estimates in GAinS and MARS; Cor = Pearson correlation, p = correlation p value. (**D**) Immune-relevant pathways enriched in SRS1 and SR2-associated genes. (**E**) 28-day survival by SRS; p = log-rank test p value. (**F**) Proportion of patients with septic shock; p = Fisher's test p value. (**G**) Proportion of patients with acute kidney injury, with severity rated using AKI scores; p = Fisher's test p value. (**H**) Association between organ-specific cardiovascular SOFA scores and SRS; p = Mood's test p value. (**I**) Association between SRSq and total SOFA scores; R = Pearson correlation, p = correlation p value. (**J**) 28-day survival by SRS and Mars combinations; p = log-rank test p value. (**K**) Causal model used for mediation analysis. (**L**) Mediation analysis results, with solid and dotted lines showing 95% confidence intervals for the treatment (high SRSq) and control (low SRSq) conditions; ACME = Average Causal Mediation Effect; ADE = Average Direct Effect; p = mediation p value.

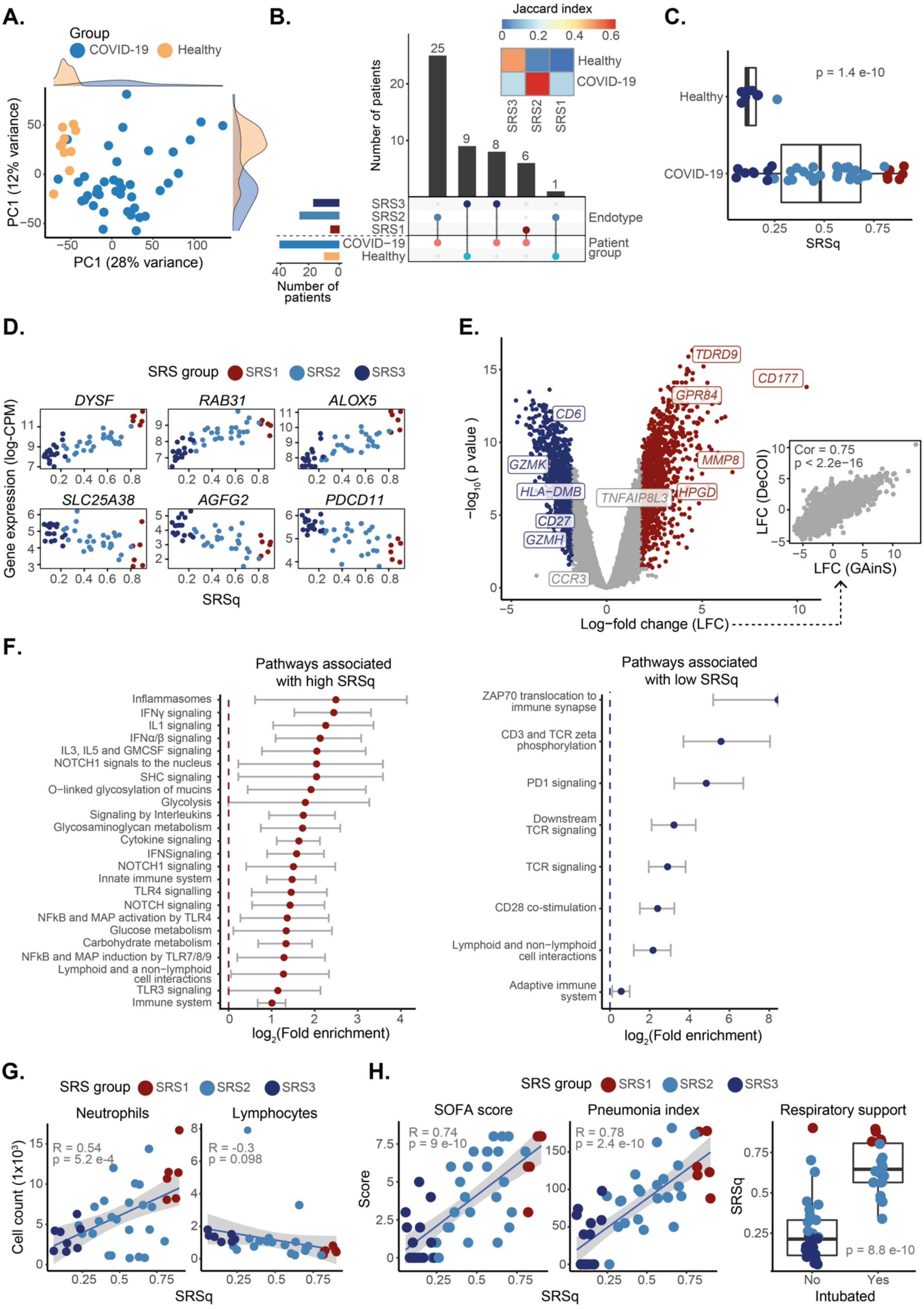

Fig. S7. SRSq validation in the DeCOI study. (**A**) Whole transcriptome PCA plot colored by clinical group. (**B**) UpSet plot (bottom) showing the overlap between SRS and clinical groups.

The heatmap (top) indicates the degree of these overlaps (i.e. Jaccard indexes). (**C**) SRSq stratified by clinical group. Samples are colored by SRS group (red = SRS1, light blue = SRS2, dark blue = SRS3); p = Kruskal-Wallis test p value. (**D**) Example genes positively and negatively associated with SRSq. Samples are colored by SRS. (**E**) Volcano plot of SRSq-associated genes. Red indicates positive and blue negative associations. The scatter plot (right) shows effect sizes in GAinS and DeCOI; Cor = Pearson correlation coefficient, p = correlation p value. (**F**) Immune-relevant pathways enriched for genes positively (left) or negatively (right) associated with SRSq at FDR < 0.05. (**G**) Association between SRSq and cell counts (Z-scored). Samples are colored by SRS group. Lines indicate best linear fits and 95% confidence intervals; Cor = Pearson correlation, p = correlation p value. (**H**) Association between SRSq and clinical variables. Samples are colored by SRS group. Lines indicate best linear fits and 95% confidence intervals; Cor = Pearson correlation, p = correlation p.

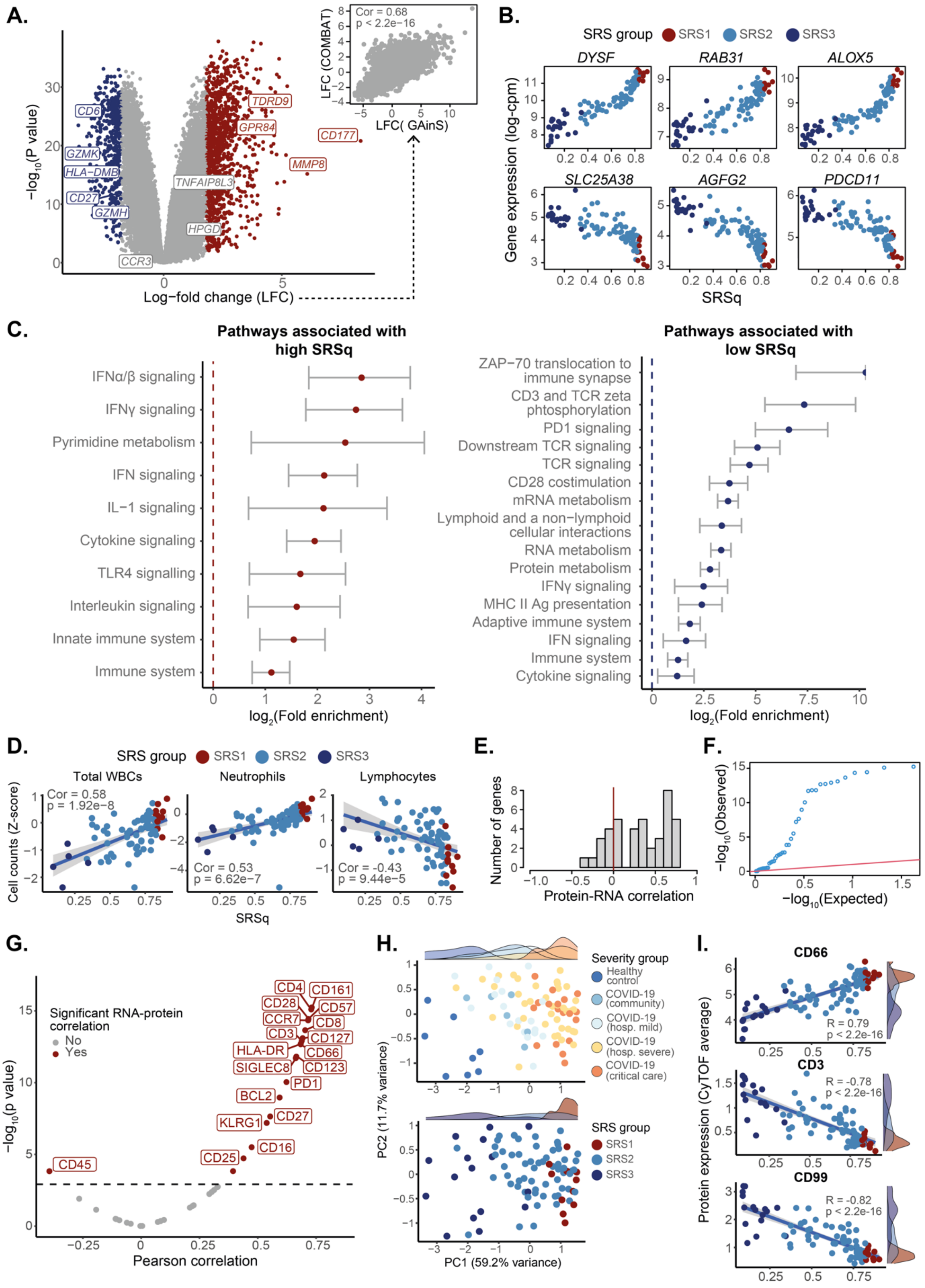

Fig. S8. Transcriptional profiles associated with SRS or SRSq in the COMBAT study. (**A**) Volcano plot showing SRSq-associated genes. Red indicates a positive and blue a negative association with SRSq. Gene names are provided for a subset of significant genes with immune

relevance. The scatter plot (right) shows the agreement between log-fold changes in sepsis (GAinS) and COVID-19 (COMBAT). Cor = Pearson correlation; p = correlation p value. (**B**) Expression pattern of example genes positively (top) and negatively (bottom) associated with SRSq. Samples are colored by SRS group. (**C**) A subset of immune-relevant pathways significantly enriched for genes differentially expressed between SRS groups. Both SRS1 (left) and SRS2-associated (right) pathways at an FDR < 0.05 are shown. Whiskers indicate 95% confidence intervals. (**D**) Association between SRSq and cell counts (Z-scored). Samples are colored by SRS group. Lines indicate best linear fits. Cor = Pearson correlation coefficient; p = correlation p value. (**E**) Histogram showing the distribution of Pearson correlation coefficients for mRNA-protein pairs across samples, as calculated based on RNA-seq and CyTOF measurements. (**F**) Quantile-quantile plot showing the distribution of mRNA-protein correlation p values. Each dot represents a gene, with the red line indicating the expectation under a null (uniform) distribution. (**G**) Scatter plot of mRNA-protein Pearson correlation coefficients (x axis) and their associated $\log_{10}$ p values (y axis). Each dot represents a gene, with dark red indicating a significant correlation between assays. The dotted line indicates the significance threshold of the Bonferroni correction for multiple testing. (**H**) Principal component analysis plot based on 40 proteins measured with CyTOF. Each dot represents a sample, colored by clinical phenotype (top) or SRS group (bottom) as inferred from RNA-seq. (**I**) Correlation between SRSq (x axis) and protein levels (y axis) for the three proteins most significantly associated with SRSq. Cor = Pearson correlation; p = correlation p value.

SUPPLEMENTARY TABLES

Table S1. Publicly available data sets used for model training and validation. List of all publicly available gene expression data sets used throughout this study, either for model training or for validation of results. All data sets consisted of whole blood transcriptomes.

Study	Data repository	Clinical phenotype	Sample size	Profiling strategy	Usage
Genomic Advances in Sepsis (GAinS)	ArrayExpress: E-MTAB-4421, E-MTAB-4451, E-MTAB-5273, E-MTAB-5274	Sepsis secondary to CAP or FP	676 samples (514 patients; 383 CAP and 131 FP)	LeukoLOCK + Illumina HumanHT-12 v4 arrays	Model training and testing
GAinS	European Genome-Phenome Archive (EGA): EGAD00001008730	Sepsis secondary to CAP or FP	864 samples (667 patients; 439 CAP and 228 FP)	LeukoLOCK + polyA RNA-seq	Model training and testing
GAinS	Data will be provided as a supplementary table upon publication	Sepsis secondary to CAP	115 samples (107 CAP patients)	LeukoLOCK + qRT-PCR	Model training and testing
Dietary, Lifestyle, and Genetic determinants of Obesity and Metabolic syndrome (DILGOM)	ArrayExpress: E-TABM-1036	Healthy volunteers	518 samples	PAXgene tubes + Illumina HumanHT-12 v3 arrays	Model training and testing
Study of Health in Pomerania (SHIP-TREND)	GEO: GSE36382	Healthy volunteers	991 samples	PAXgene tubes + Illumina HumanHT-12 v3 arrays	Model training and testing
400FG (Human Functional Genomics Project; HFGP)	GEO: GSE134080	Healthy volunteers	100 samples	PAXgene tubes + polyA RNA-seq	Model training and testing

Parnell et al. *(21)*	GEO: GSE54514	Bacterial sepsis	163 samples (36 patients, 18 controls)	PAXgene tubes + Illumina HumanHT-12 v3 arrays	Validation
Molecular Diagnosis and Risk of Sepsis (MARS)	GEO: GSE65682	All-cause Sepsis	802 samples	PAXgene tubes + HG U219 Affymetrix arrays	Validation
Wong et al. *(11)*	GEO: GSE13904	Pediatric SIRS, sepsis, and septic shock	227 samples from 152 participants (23 SIRS, 38 sepsis, 73 septic shock, 18 controls)	PAXgene tubes + U133 plus 2.0 Affymetrix arrays	Validation
Mechanisms of Severe Acute Influenza Consortium (MOSAIC)	GEO: GSE111368	H1N1 influenza	358 samples (109 patients, 130 controls)	Tempus tubes + Illumina HumanHT-12 v4 arrays	Validation
Deutsche COVID-19 Omics Initiative (DeCOI)	GitHub:<br><br>https://github.com/schultzelab/COVID-19-blood-bulk-RNA-Seq	COVID-19	49 samples<br><br>(39 patients and 10 controls)	PAXgene tubes + polyA RNA-seq	Validation
COVID-19 Multi-Omic Blood Atlas (COMBAT)	Zenodo:<br><br>https://doi.org/10.5281/zenodo.6120249	COVID-19	101 samples<br><br>(77 COVID-19 patients and 10 controls)	Tempus tubes + total RNA-seq	Validation

Table S2. Clinical variables in the GAinS study. List of clinical and outcome variables available for the GAinS cohort and used throughout this study. Different columns specify how these variables were measured and in which units.

Clinical variable	Definition	Variable type	Unit
Mortality event	Information on whether the patient died (1) or survived (0)	Binary	Yes/No
Time to mortality event	Number of days from ICU admission to death (if it occurred)	Numeric (integer)	Days
Cell counts	Full blood counts as measured in hospital using hematology analyzers	Numeric (float)	$1x10^9$ cells/l
APACHE scores	Total Acute Physiology and Chronic Health Evaluation (APACHE) II scores measured at ICU admission	Numeric (integer)	0-24 points
Total SOFA scores	Total Sequential Organ Failure Assessment (SOFA) scores measured during the first, third and/or fifth days after ICU admission	Numeric (integer)	0-71 points
Mean arterial pressure (MAP)	Lowest mean arterial pressure measurement obtained for each patient	Numeric (float)	mmHg
PF ratio	PaO_2/FiO_2 ratio (i.e. Horowitz index)	Numeric (float)	mmHg
Bilirubin concentration	Highest bilirubin measurement obtained for each patient	Numeric (float)	µmol/l
Creatinine concentration	Highest creatinine measurement obtained for each patient	Numeric (float)	µmol/l
Platelet counts	Number of platelets measured	Numeric (float)	$1x10^3$ platelets/µl
ICU-AI scores	ICU-acquired infection score.<br><br>This score reflects how many of the following ICU-acquired complications were diagnosed for each patient: 1) ventilator associated pneumonia (VAP), 2) lower respiratory tract infection, 3) bacteremia, 4) line-related infection, 5) wound infection, 6) urinary tract infection (UTI), 7) other infection	Ordinal (integer)	0-7 points

Table S3. Primers used for qRT-PCR. Characteristics of primers used for qRT-PCR amplification, including sequence, target gene, and amplicon size.

Gene name	RefSeq ID	Primer sequence<br>(Forward and Reverse; 5’ to 3’)	Amplicon length (bp)
ACTB	NM_001101.2	F – AAAAACTGGAACGGTGAAGGTGAC<br>R – CCTGTAACAACGCATCTCATATTTGG	136
ADGRE3	NM_032571.4	F – AAAACCCAGTGAGGGGGATG<br>R – GAGAGCCTATTGTGGAGAACAA	214
ARL14EP	NM_152316.3	F – TTCAGACAGACAAGTGATACCAGCAAA<br>R – ACCGCAGATTATCCAGCATGT	246
CCNBP1IP1	NM_182852.1	F – TGGAGCGCAATCGTCAGTAT<br>R – AGTTGTTACCTAATGGGAAGCCA	147
DYRK2	NM_003583.2	F – AGTAAGGCCAATGTTAACAACACG<br>R – CCAAACTCTCAGTTACTTTGCCAG	193
MDC1	NM_014641.3	F – CGGTCCTATAAGCCTCAGAGAGTT<br>R – TCTTCTAATTCGTGGTCTGGGAG	223
TDRD9	NM_153046.1	F – CCCTACGAGTGGAATCAGGTTG<br>R – CCTTTAGGATGCAGTGGGCA	247
TOP1	NM_003286.2	F – CTCTGAGAGCAGGCAATGAAAAG<br>R – CTCTGGGTGTAGATTGATGTGCT	95
ZAP70	NM_207519.1	F – CATGGACACGAGCGTGTATGAG<br>R – CCACGTCGATCTGCTTCTTGC	173
